# Inhaled diesel exhaust particles result in microbiome-related systemic inflammation and altered cardiovascular disease biomarkers in C57Bl/6 male mice

**DOI:** 10.1186/s12989-022-00452-3

**Published:** 2022-02-09

**Authors:** Danielle T. Phillippi, Sarah Daniel, Vaidehi Pusadkar, Victoria L. Youngblood, Kayla N. Nguyen, Rajeev K. Azad, Brian K. McFarlin, Amie K. Lund

**Affiliations:** 1 Department of Biological Sciences, Advanced Environmental Research Institute, University of North Texas, EESAT – 215, 1704 W. Mulberry, Denton, TX 76203 USA; 2 BioDiscovery Institute, Department of Biological Sciences, University of North Texas, Denton, TX 76203 USA; 3 Department of Biological Sciences, University of North Texas, Denton, TX 76203 USA; 4 Department of Mathematics, University of North Texas, Denton, TX 76203 USA; 5 UNT Applied Physiology Laboratory, University of North Texas, Denton, TX 76203 USA

**Keywords:** Diesel particulate matter, Gut microbiome, Probiotics, Cardiovascular disease, Inflammation, High fat diet, Endotoxin, Bifidobacterium, Lactobacillus, Lactococcus

## Abstract

**Background:**

The gut microbiota plays a vital role in host homeostasis and is associated with inflammation and cardiovascular disease (CVD) risk. Exposure to particulate matter (PM) is a known mediator of inflammation and CVD and is reported to promote dysbiosis and decreased intestinal integrity. However, the role of inhaled traffic-generated PM on the gut microbiome and its corresponding systemic effects are not well-characterized. Thus, we investigated the hypothesis that exposure to inhaled diesel exhaust particles (DEP) alters the gut microbiome and promotes microbial-related inflammation and CVD biomarkers. 4–6-week-old male C57Bl/6 mice on either a low-fat (LF, 10% fat) or high-fat (HF, 45% fat) diet were exposed via oropharyngeal aspiration to 35 μg DEP suspended in 35 μl saline or saline only (CON) 2x/week for 30 days. To determine whether probiotics could prevent diet or DEP exposure mediated alterations in the gut microbiome or systemic outcomes, a subset of animals on the HF diet were treated orally with 0.3 g/day (~ 7.5 × 10^8^ CFU/day) of Winclove Ecologic® Barrier probiotics throughout the study.

**Results:**

Our results show that inhaled DEP exposure alters gut microbial profiles, including reducing Actinobacteria and expanding Verrucomicrobia and Proteobacteria. We observed increased circulating LPS, altered circulating cytokines (IL-1α, IL-3, IL-13, IL-15, G-CSF, LIF, MIP-2, and TNF-α), and CVD biomarkers (siCAM, PAI-1, sP-Selectin, thrombomodulin, and PECAM) in DEP-exposed and/or HF diet mice. Furthermore, probiotics attenuated the observed reduction of Actinobacteria and expansion of Proteobacteria in DEP-exposed and HF-diet mice. Probiotics mitigated circulating cytokines (IL-3, IL-13, G-CSF, RANTES, and TNF- α) and CVD biomarkers (siCAM, PAI-1, sP-Selectin, thrombomodulin, and PECAM) in respect to DEP-exposure and/or HF diet.

**Conclusion:**

Key findings of this study are that inhaled DEP exposure alters small intestinal microbial profiles that play a role in systemic inflammation and early CVD biomarkers. Probiotic treatment in this study was fundamental in understanding the role of inhaled DEP on the microbiome and related systemic inflammatory and CVD biomarkers.

**Supplementary Information:**

The online version contains supplementary material available at 10.1186/s12989-022-00452-3.

## Introduction

The gut microbiota is a dynamic group of microorganisms that plays a pivotal role in the homeostasis of its host by providing nutrients, providing protection from infections, stimulating the immune system, and aiding in digestion, among others [1]. The microbial communities in the intestines are constantly adapting to environmental and dietary changes of the host [2]. Exposure to air pollutants, such as particulate matter (PM), has been shown to alter the gut microbiome composition, increase the risk of obesity, gastrointestinal disorders, inflammation, cardiovascular disease (CVD), and other chronic illnesses [3–9]. Early epidemiological studies have shown an increased incidence of hospitalization on days of high air pollution due to gastrointestinal distress, such as inflammatory bowel disease, ulcerative colitis, and Chron’s disease [10–12]. Collectively, these correlations emphasize a need to investigate further and characterize the effects of PM on the gut microbiome. Several studies have identified the effects of ingested PM on the gut microbiome; however, relatively few studies have aimed to understand the effects of inhaled PM on the gut microbiome [13–15]. Recent studies have shown that inhaled ambient PM exposure resulted in dysbiosis in the gut and intestinal inflammation in mice [9, 10, 15]. Furthermore, we have previously reported that whole-body inhalational exposure to traffic-generated emissions and wood smoke resulted in alterations in the gut microbiome and increased intestinal permeability in ApoE^−/−^ mice [16]. With a more recent exploration of inhaled PM on the microbiome, there is a need to understand further the mechanisms by which PM alters the microbiome and the resulting physiological effects.

CVD is the leading cause of morbidity and mortality globally, with air pollution being one of the greatest non-genetic contributing factors [7]. Exposure to PM is associated with an increased risk of morbidity and mortality from cardiovascular events such as myocardial infarction, stroke, arrhythmias, and heart failure [6]. Established mechanisms by which inhaled PM affects the heart include inflammation, oxidative stress, systemic cytokine cascades, and pathophysiologic vascular outcomes via interactions through the circulation [6, 17, 18]. In addition to environmental factors, diet and obesity also contribute to the etiology of CVD. Many people in the United States consume diets high in fats and cholesterol, often referred to as a Western diet, contributing to obesity and CVD [19]. Measurement of biomarkers for vascular endothelial dysfunction and immune cell modulation is important in the early detection of CVD. Cell adhesion molecules, such as platelet endothelial cell adhesion molecule (PECAM), intercellular adhesion molecule-1 (ICAM-1), and soluble platelet- selectin (sP-selectin) mediate blood cell-endothelial cell interactions during pathological and physiological conditions by immune cell recruitment and leukocyte-endothelial cell adhesion and are implicated in CVD [20, 21]. Platelet activator inhibitor-1 (PAI-1) is an important mediator in fibrinolytic activity during endothelial damage and is also strongly induced during endotoxemia [22]. Thrombomodulin is clinically indicated in CVD because of its role as a thrombin-inhibitor resulting in anticoagulant anti-inflammatory and leukocyte adhesion inhibitor effects [23].

Previous studies have reported an association between CVD and gut microbiome profiles. One comprehensive study found decreased *Bacteroides*, *Ruminococcus*, and *Prevotella* and increased *Streptococcus* and *Escherichia* with atherosclerotic CVD [24]. It has also been shown that *Clostridium* and *Streptococcus* are increased in hypertensive patients, while *Bifidobacterium* is reduced [25]. There also is a clear connection between the gut microbiome and systemic inflammation. Functional groups of the microbiota can contribute to cytokine production, such as tumor necrosis factor-α (TNF-α), which is inversely proportional with *Bifidobacterium* presence [26]. Lipopolysaccharide (LPS) toxin is present in the cell wall of gram-negative bacteria and is an important mediator of local and systemic inflammation [27]. Disruption in the intestinal epithelial barrier due to stress is thought to be the primary source of systemic LPS [28]. LPS has been shown to activate local and systemic toll-like receptors (TLRs) and increase systemic levels of inflammatory cytokines and chemokines, which are indicated in chronic heart disease [29–31]. The expression of pro- and anti-inflammatory cytokines is often associated with physiological stress, including toxic exposures, disease-states, and microbial-derived LPS [26, 32, 33]. Furthermore, high-fat (HF) diet consumption has also been associated with alterations in the gut microbial composition and a decrease in intestinal integrity [34]. Multiple studies have shown that a HF diet alters the gut microbiome, induces gut-derived endotoxemia and systemic inflammation [35, 36].

Probiotics are living microorganisms intended to benefit overall health by contributing to the function of the gut microbiome, providing essential metabolites, competing with pathogens, and managing the microbial profiles in the gut. The recent expansion of metagenomic technology has provided insight into the use of probiotic treatment in treating diseases and efficacy within the microflora. Probiotics often contain bacteria such as gram-positive bacteria *Lactobacillus* or *Bifidobacterium* or yeast such as *Saccharomyces.* Probiotics using *Lactobacillus* strains have been shown to decrease cholesterol, reduce atherosclerosis, and have cardioprotective and anti-inflammatory effects [37–39]. Probiotics using *Bifidobacterium* strains have also been shown to reduce LPS, stimulate immune cell modulation and decrease cytokine concentrations [37, 40, 41]. The ability of probiotics to alter the profile of the gut microbiome is of particular interest for understanding its contribution to health and disease, especially during environmental exposure to toxins.

We have previously reported alterations in microbial profiles and local inflammation in the lungs resulting from diesel PM exposure combined with a HF diet, which were mitigated through probiotic-treatment. While the effects of inhaled traffic-generated PM and HF diet on the gut and CVD have been explored, to our knowledge, there are no studies that investigate the effects on the gut microbiome and associated expression of CVD biomarkers that include probiotic treatment. Understanding the role of the gut microbiome in promoting systemic inflammation and CVD following PM exposure is vital in the future development and application of microbiome-related therapeutics and interventions in health. Considering this gap in knowledge, we investigated the hypothesis that exposure to inhaled diesel exhaust PM (DEP) results in alterations in gut microbiota profiles, systemic inflammation, and CVD risk, which is exacerbated by HF diet consumption in male C57Bl/6 mice. Furthermore, we investigated whether the observed exposure and diet-mediated outcomes could be mitigated through probiotic treatment.

## Results

### Exposure to inhaled DEP and high-fat diet alters small intestinal microbial profiles and diversity in C57Bl/6 mice

**Fig. 1:**
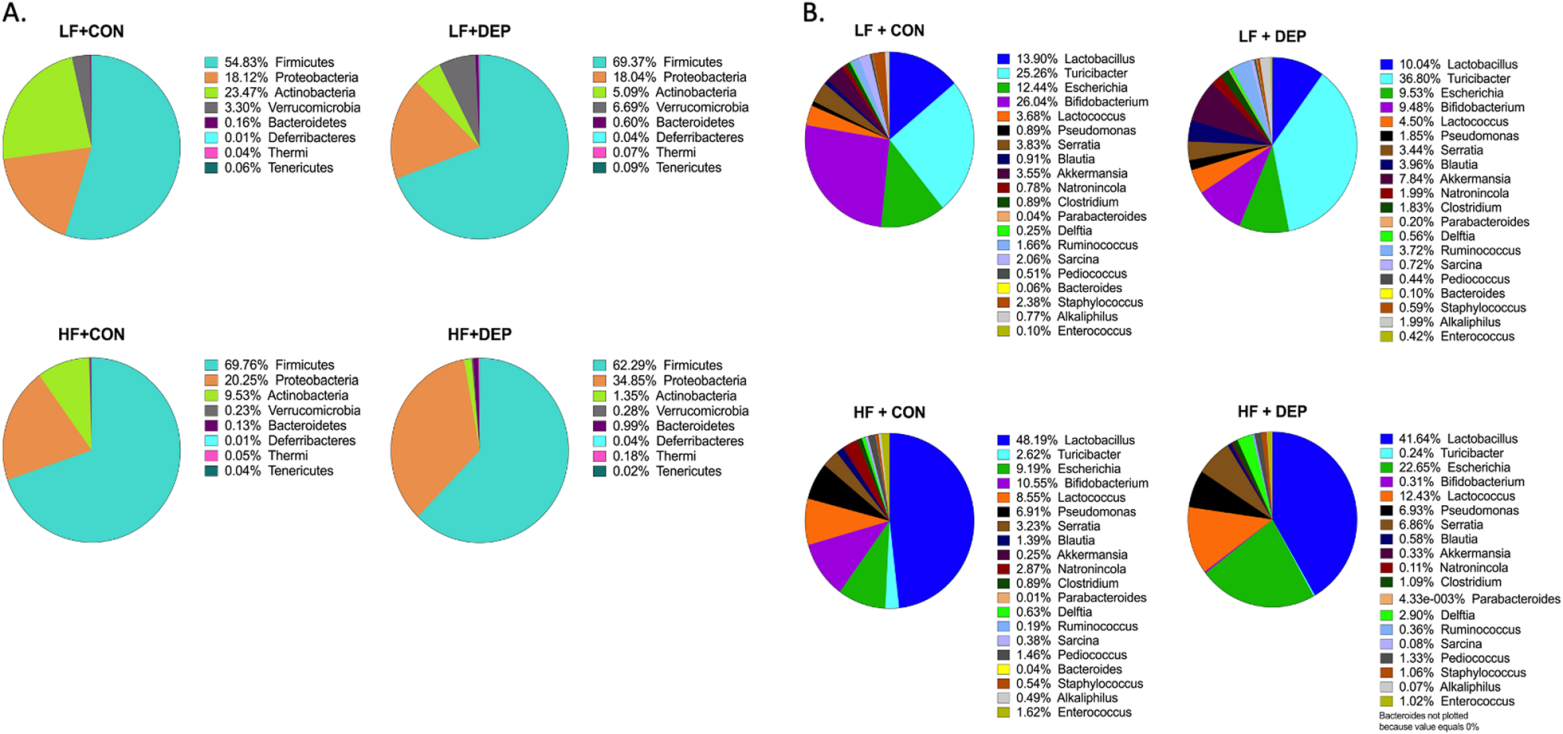
Exposure to inhaled DEP results in decreased relative abundance of Actinobacteria and expansion of Proteobacteria. Data shows normalized 100% relative abundance for **A** upper eight most abundant phyla pie chart with percentages and the **B** upper 20 most abundant genus pie chart with percentages within the small intestine of C57Bl/6 male mice exposed to either diesel exhaust particles (DEP- 35 μg PM) or saline (CON) twice a week for four weeks on either low-fat (LF) or high-fat (HF) diet. Top phyla and genera relative abundances were determined and analyzed in regards to respective control. *n* = 6–7

**Fig. 2:**
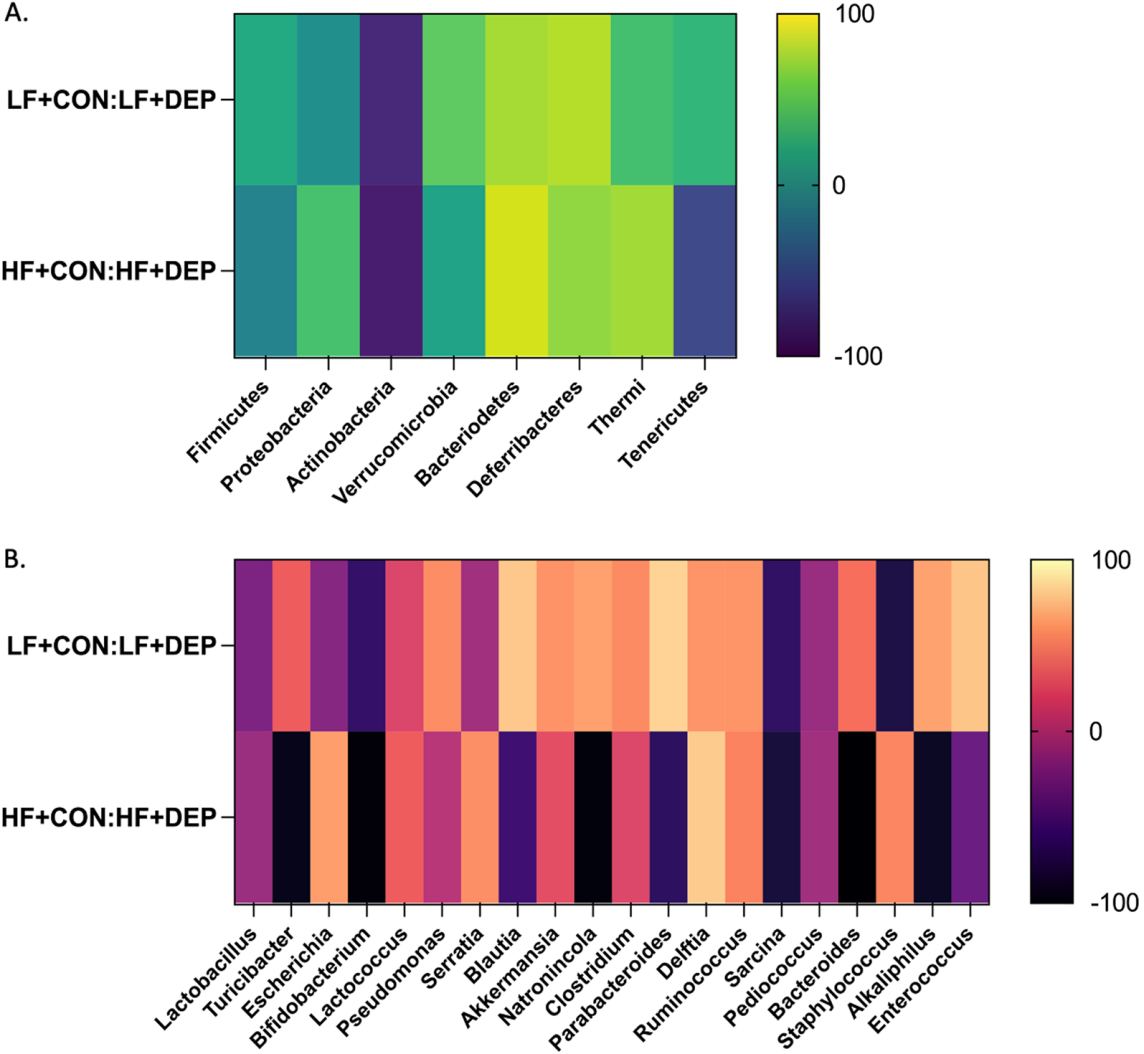
Exposure to inhaled DEP results in changes in microbial profiles which is exacerbated in high-fat fed mice. Heat map shows the percent change in DEP with their respective HF and LF controls for most abundant **A** phyla and **B** genus in C57Bl/6 male mice exposed to inhaled diesel exhaust particles (DEP- 35 μg PM) compared to saline control (CON) twice a week for four weeks on either low-fat (LF) or high-fat (HF) diet. An increase in bacteria with DEP exposure is indicated by a positive percentage and a decrease in bacteria with DEP with exposure is indicated by a negative percentage. Values can be viewed in Table 1

To understand the effects of inhaled DEP on the gut microbial profiles, we determined the relative abundance of bacteria in the small intestine of male C57Bl/6 mice exposed to inhaled DEP or saline controls (CON) fed a low-fat (LF) or high-fat (HF) diet. Figure 1 shows the relative abundance and relative percentage out of 100% of the top eight (A) phyla or top twenty (B) genus present in the small intestine in these animals. Additional file 1: Table S1 shows the mean, standard deviation, and standard error of mean for each group at the phyla level. To represent the changes in microbial profiles in response to inhaled DEP exposure, we used the average relative abundance percentages at the phyla and genus level and calculated the percent change compared to saline-control in their respective LF and HF group (Fig. 2). DEP exposure in the LF diet animals resulted in a 20.96% increase in Firmicutes; however, DEP exposure in the HF diet animals resulted in a 10.70% decrease in the Firmicutes. We also observed a small reduction in Proteobacteria with DEP exposure in the LF animals; however, we observed an expansion of Proteobacteria by 41.9% in HF + DEP mice, compared to HF + CON. Within the Proteobacteria phyla, DEP exposure was associated with an increase in Pseudomonas, regardless of diet, compared to their respective controls. DEP exposure mediated a decrease in *Escherichia* by 23.43% in the LF diet animals; conversely, DEP exposure mediated an increase of *Escherichia* by 59.41% in HF diet animals, compared to their respective controls. We found an overall reduction in Actinobacteria and an overall increase in Verrucomicrobia in the small intestines of DEP-exposed mice. Additionally, we observed an increase in Bacteroidetes with exposure to DEP, which was further increased with consumption of a HF diet. Table 1 shows the percent change in relative abundance and difference in average percent relative abundance resulting from DEP exposure in both the LF and HF groups, compared to their respective controls, which is represented in Fig. 2. The genus has been categorized into its respective phyla.

**Table 1:** Percent change in most abundant phyla and genus across exposure and diet groups

	LF + DEP compared to LF + CON		HF + DEP compared to HF + CON	
	Percent change (%)	Difference	Percent change (%)	Difference
*P_Firmicutes*	+ 20.96	+ 14.54	− 10.70	− 7.47
G_Lactobacillus	− 27.80	− 3.87	− 13.60	− 6.56
G_Turicibacter	+ 31.35	+ 11.54	− 90.69	− 2.38
G_Lactococcus	+ 18.22	+ 0.82	+ 31.24	+ 3.88
G_Blautia	+ 76.98	+ 3.05	− 58.60	− 0.82
G_Natronincola	+ 60.76	+ 1.21	− 96.03	− 2.76
G_Clostridium	+ 51.45	+ 0.94	+ 18.63	+ 0.20
G_Ruminococcus	+ 55.37	+ 2.06	+ 48.16	+ 0.18
G_Sarcina	− 65.14	− 1.34	− 78.41	− 0.29
G_Pediococcus	− 14.23	− 0.07	− 9.90	− 0.13
G_Staphylococcus	− 75.17	− 1.79	+ 49.04	+ 0.52
G_Alkalphilius	+ 61.25	+ 1.22	− 86.51	− 0.42
G_Enterococcus	+ 75.97	+ 0.32	− 36.91	− 0.60
*P_Proteobacteria*	− 0.45	− 0.08	+ 41.9	+ 14.60
G_Escherichia	− 23.43	− 2.91	+ 59.41	+ 13.46
G_Pseudomonas	+ 52.21	+ 0.97	+ 0.34	+ 0.02
G_Serratia	− 10.07	− 0.39	+ 52.98	+ 3.64
G_Delftia	+ 55.41	+ 0.31	+ 78.39	+ 2.27
*P_Actinobacteria*	− 78.32	− 18.38	− 85.81	− 8.18
G_Bifidobacterium	− 63.59	− 16.56	− 97.08	− 10.24
*P_Verrucomicrobia*	+ 50.71	+ 3.39	+ 16.06	+ 0.04
G_Akkermansia	+ 54.76	+ 4.30	+ 23.06	+ 0.08
*P_Bacteriodetes*	+ 73.36	+ 0.44	+ 86.91	+ 0.86
G_Parabacteroides	+ 81.81	+ 0.16	− 66.75	− 0.01
G_Bacteroides	+ 39.62	+ 0.04	− 100.00	− 0.04
P_Deferribacteres	+ 77.94	+ 0.03	+ 67.23	+ 0.03
P_Thermi	+ 40.38	+ 0.03	+ 72.50	+ 0.13
P_Tenericutes	+ 32.11	+ 0.03	− 55.75	− 0.02

DEP, diesel exhaust particles; CON, saline controls; LF, low fat diet; HF, high fat diet

**Fig. 3:**
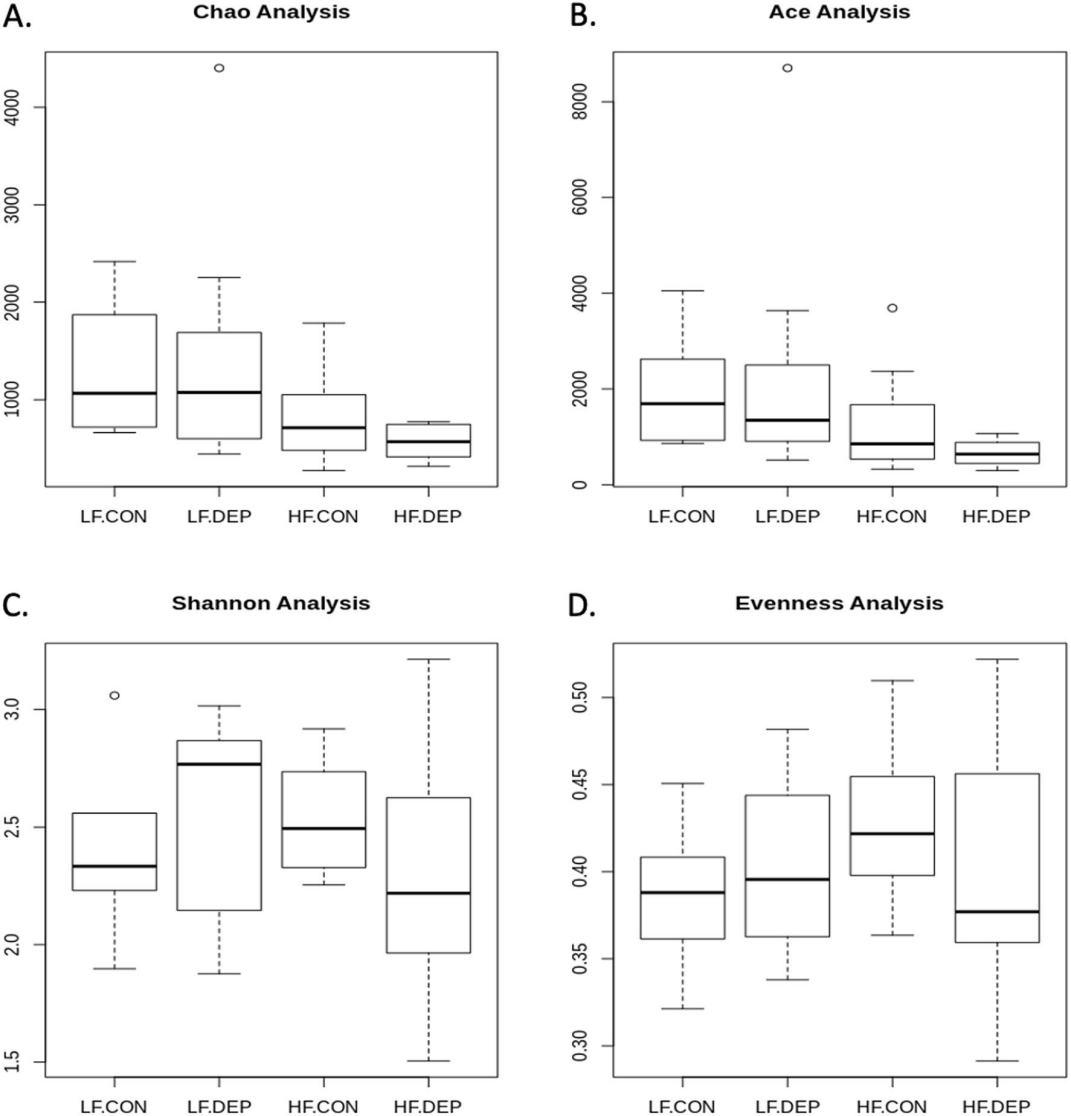
Bacterial alpha diversity analysis between exposure and diet. Alpha diversity analysis using **A** Chao, **B** Ace, **C** Shannon, and **D** Evenness compared in C57Bl/6 male mice exposed to inhaled diesel exhaust particles (DEP- 35 μg PM) or saline control (CON) twice a week for four weeks on either low-fat (LF) or high-fat (HF) diet. *n* = 6–7

**Fig. 4:**
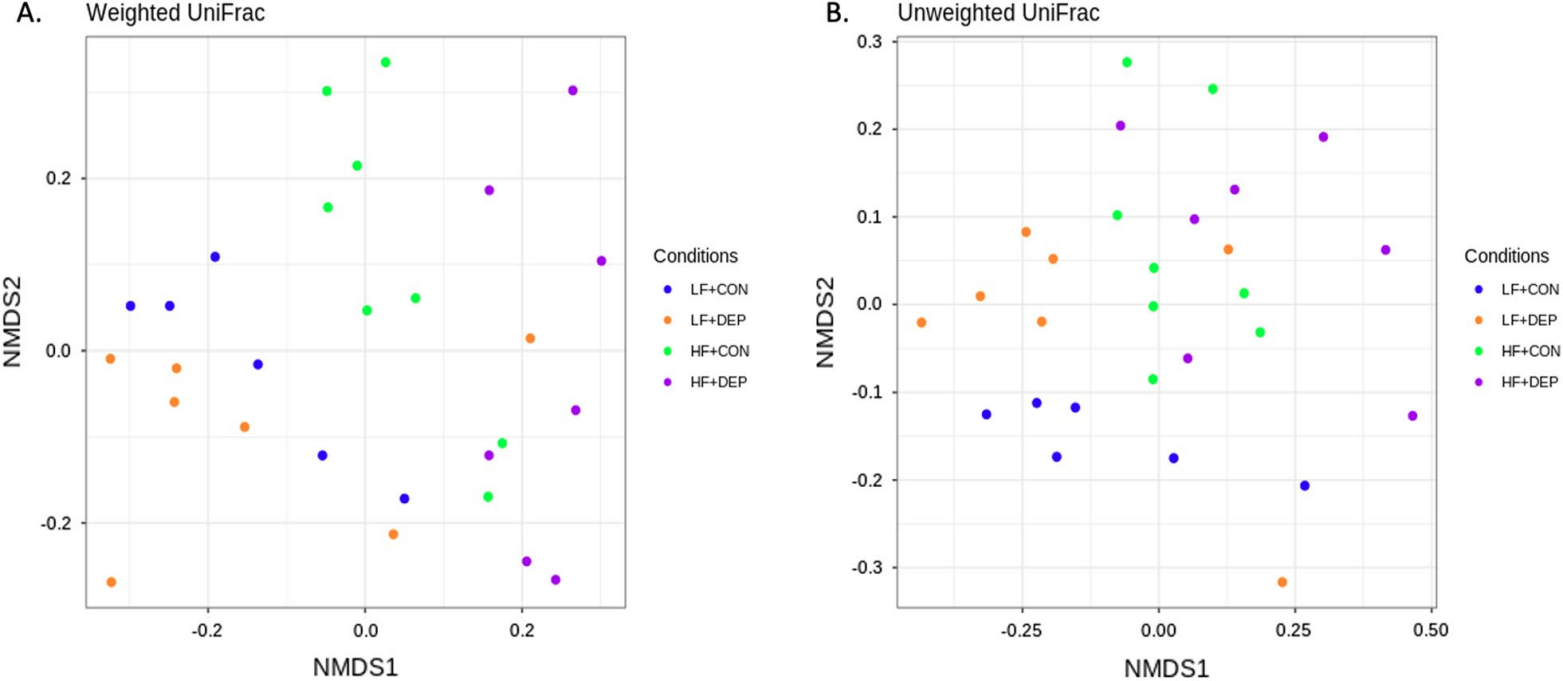
Bacterial beta diversity analysis between exposure and diet. Beta diversity was calculated using UniFrac distances and Principle Coordinates Analysis to show the similarities in microbial communities in both **A** weighted and **B** unweighted analyses. Each dot represents compared in C57Bl/6 male mice exposed to inhaled diesel exhaust particles (DEP- 35 μg PM) or saline control (CON) twice a week for four weeks on either low-fat (LF) or high-fat (HF) diet. *n* = 6–7

We calculated the α-diversity of microbial profiles in the small intestine, as determined by richness (Chao1 and ACE) and diversity (Shannon, Evenness) (Fig. 3). We observed a decrease in richness and an increase in diversity with DEP exposure in the LF diet animals, compared to controls. However, there was a decrease in both richness and diversity with DEP exposure between HF diet animals, compared to controls. There were no statistical differences in α-diversity with exposure, diet, or interaction by ANOVA; however, there were observed statistical differences in β-diversity in microbial profiles within the small intestine between these four groups. Both weighted (Fig. 4A) and unweighted (Fig. 4B) PCoA plots show dispersing of the groups concerning diet, but not DEP exposure. An Analysis of Molecular Variance (AMOVA) showed UniFrac distances with significant differences for comparisons between all four groups (LF + CON:HF + CON:LF + DEP:HF + DEP) with *p*-value of < 0.001, between LF + CON and HF + CON groups with *p*-value of < 0.001, between LF + CON and HF + DEP groups with *p*-value of 0.005, between LF + DEP and HF + DEP groups with *p*-value of 0.002 (Table 2).

**Table 2:** AMOVA analysis of microbial profiles from small intestine

Exposure groups	SS	Df	MS	Fs	*p*-value
*HF* + *CON * versus* HF* + *DEP * versus* LF* + *CON * versus* LF* + *DEP*				3.64601	< 0.001
Among	2.46128	3	0.820428		
Within	5.4005	24	0.225021		
Total	7.86178	27			
*HF* + *CON * versus* HF* + *DEP*				1.25701	0.276
Among	0.290038	1	0.290038		
Within	2.99957	13	0.230737		
Total	3.28961	14			
*HF* + *CON * versus* LF* + *CON*				3.9775	0.002
Among	0.821598	1	0.821598		
Within	2.47874	12	0.206561		
Total	3.30033	13			
*HF* + *CON * versus* LF* + *DEP*				5.3469	0.001
Among	1.21957	1	1.21957		
Within	2.96515	13	0.228088		
Total	4.18472	14			
*HF* + *DEP * versus* LF* + *CON*				4.61211	0.005
Among	1.0211	1	1.0211		
Within	2.43535	11	0.221395		
Total	3.45645	12			
*HF* + *DEP * versus* LF* + *DEP*				4.71885	0.002
Among	1.14895	1	1.14895		
Within	2.92176	12	0.24348		
Total	4.07071	13			
*LF* + *CON * versus* LF* + *DEP*				1.90662	0.098
Among	0.41615	1	0.41615		
Within	2.40092	11	0.218266		
Total	2.81707	12			

DEP, diesel exhaust particles; CON, saline controls; HF, high-fat diet; LF, low-fat diet; SS, sum of squares; Df; degrees of freedom; MS, mean of squares

### Exposure to inhaled DEP and high-fat diet increases circulating LPS in male C57Bl/6 mice

**Fig. 5:**
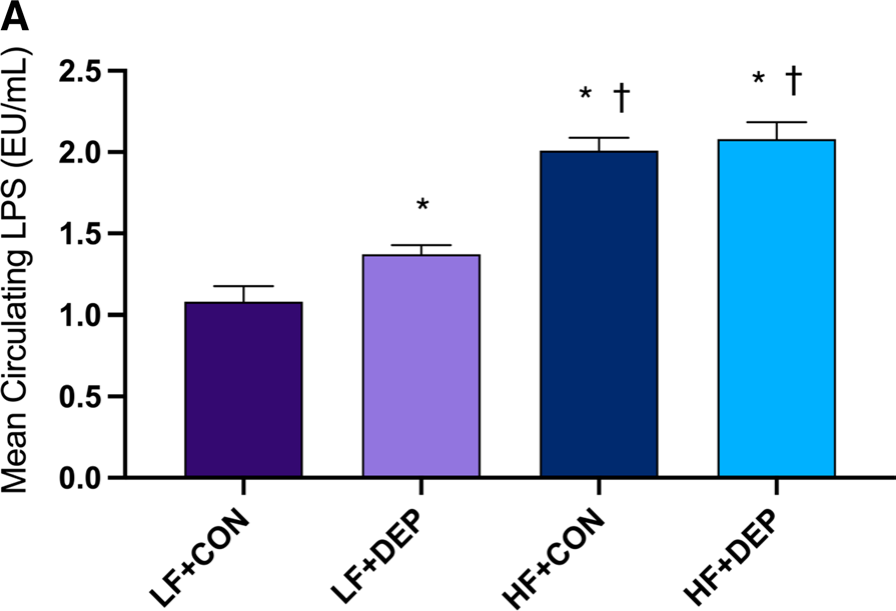
Exposure to inhaled DEP and high-fat diet increases circulating LPS. Mean circulating LPS **A** significantly increases in C57Bl/6 male mice exposed to diesel exhaust particles (DEP- 35 μg PM) or saline control (CON) twice a week for four weeks on either low-fat (LF) or high-fat (HF) diet. Data are depicted as ± SEM with *p < 0.05 compared to LF + CON, †*p* < 0.05 compared to LF + DEP, and ‡*p* < 0.05 compared to HF + CON by two-way ANOVA

To determine if inhaled DEP exposure altered circulating LPS levels, we measured plasma LPS levels in our study animals. We observed a significant increase in LPS with DEP exposure and diet (Fig. 5). Compared to LF + CON, LPS was significantly increased in LF + DEP (*p* = 0.023), HF + CON (*p* < 0.001), and HF + DEP (*p* < 0.001) groups. Compared to LF + DEP, LPS was increased in HF + CON (*p* < 0.001) and HF + DEP (*p* < 0.001) groups. There was no observed increase in LPS for HF + DEP when compared to HF + CON (*p* < 0.5698) group. The respective F values for circulating LPS are as follows: exposure F = 4.56, diet F = 92.73, exposure X diet F = 1.7.

### Exposure to inhaled DEP alters circulating cytokine and chemokines in male C57Bl/6 mice

**Fig. 6:**
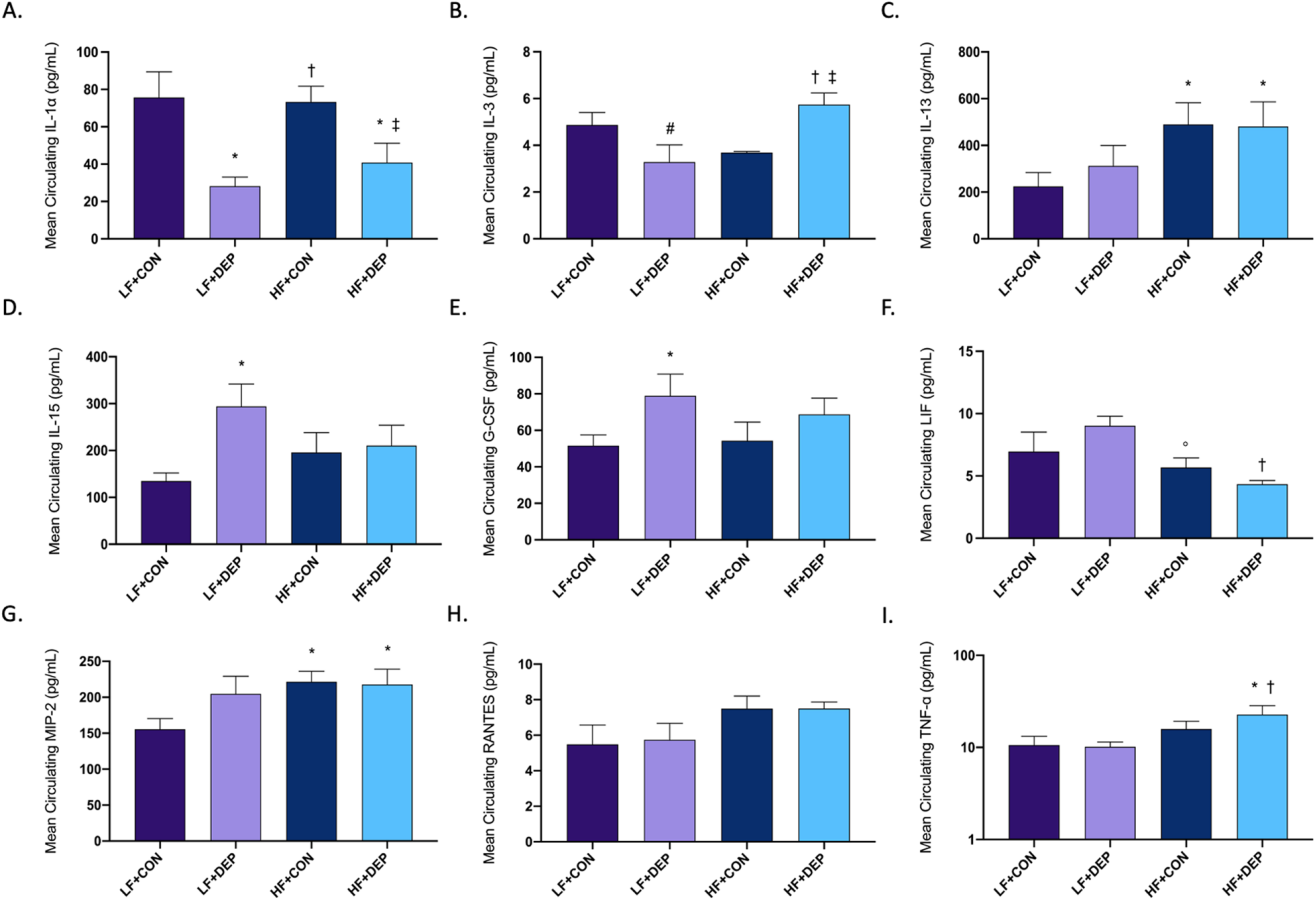
Exposure to inhaled DEP and high-fat diet alters circulating cytokine and chemokines. Mean concentration for circulating inflammatory factors: **A** IL-1α, **B** IL-3, **C** IL-13, **D** IL-15, **E** G-CSF, **F** LIF, **G** MIP-2, **H** RANTES, and **I** TNF-α measured in plasma in C57Bl/6 male mice exposed to inhaled diesel exhaust particles (DEP- 35 μg PM) or saline control (CON) twice a week for four weeks on either low-fat (LF) or high-fat (HF) diet. Data are depicted as ± SEM with *p < 0.05 compared to LF + CON, †*p* < 0.05 compared to LF + DEP, ‡*p* < 0.05 compared to HF + CON, #*p* < 0.07 compared to LF + CON, and ◦*p* < 0.06 compared to LF + DEP by two-way ANOVA

Interleukin (IL) cytokines are a large class of immunomodulatory proteins that mediate growth, differentiation, and activation of cells during immune responses [32, 42]. To determine the impact of inhaled DEP exposure ± a HF diet on systemic inflammation, we measured the concentration of ILs in the plasma. As shown in Fig. 6A, we observed a significant decrease in IL-1α with DEP exposure. Compared to their respective controls, DEP exposure is associated with a significant decrease in IL-1α in both the LF and HF groups (Fig. 6A; *p* = 0.001) and HF + DEP (*p* = 0.023) groups. The respective F values for IL-1α are as follows: exposure F = 18.57, diet F = 0.303, and exposure X diet F = 0.425. We also observed a DEP exposure mediated effect on IL-3 concentration; interestingly, we observed opposite responses in the LF and HF diet groups (Fig. 6B). Compared to LF + CON, there was a decrease in IL-3 for LF + DEP, though not statistical (*p* = 0.066); however, there was a significant increase in IL-3 for HF + DEP group compared to both LF + DEP (*p* = 0.004) and HF + CON (*p* = 0.018) groups. The respective F values for IL-3 are as follows: exposure F = 0.24, diet F = 1.91, and exposure X diet F = 15.31. For IL-13, we observe a significant increase in both the HF diet groups, regardless of DEP exposure (Fig. 6C). When compared to LF + CON, there is a significant increase in IL-13 for HF + CON (*p* = 0.034) and HF + DEP (*p* = 0.047) groups. The respective F values for IL-13 are as follows: exposure F = 0.207, diet F = 6.239, and exposure X diet F = 0.306. DEP-exposure mediated a significant increase in circulating IL-15 with exposure in the LF diet groups (Fig. 6D); however, there was no difference noted in either the HF + CON or HF + DEP animals. The respective F values for IL-15 are as follows: exposure F = 4.523, diet F = 0.077, and exposure X diet F = 3.129.

In addition to ILs, there are several cytokines involved in mediating immune response. To further understand the cytokine and chemokines response to DEP exposure ± a HF diet, we measured cytokines in the plasma. We observed a significant increase in granulocyte colony-stimulating factor (G-CSF) in LF + DEP animals, compared to LF + CON (Fig. 6E; *p* = 0.041). No alteration in the concentration of G-CSF was observed in the HF animals. The respective F values for G-CSF are as follows: exposure F = 5.065, diet F = 0.166, and exposure X diet F = 0.482. Diet-related changes for leukemia inhibitory factor (LIF) were observed in the current study (Fig. 6F). Compared to LF + DEP, there is a significant decrease in LIF for HF + DEP (*p* = 0.026) and a notable decrease for HF + CON (*p* = 0.053). The respective F values for LIF are as follows: exposure F = 0.096, diet F = 6.04, and exposure X diet F = 1.986. Conversely, we observed an increase in macrophage inflammatory protein (MIP-2) for the HF diet groups, compared to LF diet (Fig. 6G). When compared to LF + CON, there is a significant increase in MIP-2 concentration in the HF + CON (*p* = 0.022) and HF + DEP (*p* = 0.036) groups, but no statistical alterations were observed in the LF + DEP group (*p* = 0.090). The respective F values for MIP-2 are as follows: exposure F = 1.449, diet F = 4.374, and exposure X diet F = 1.943. There were no statistical alterations observed in systemic regulated upon activation, normal T cell expressed, and presumably secreted (RANTES) across any of the diet or DEP exposure groups (Fig. 6H). However, there was a significant increase in systemic TNF-α with HF diet (Fig. 6I). DEP exposure also mediated an increase in TNF-α in HF + CON when compared to LF CON and DEP groups (*p* = 0.021, *p* = 0.018, respectively). The respective F values for TNF-α are as follows: exposure F = 0.896, diet F = 6.831, and exposure x diet F = 1.151. We observed no statistical differences for interferon gamma (IFNγ), IL-1β, IL-2, IL-9, IL-12(p20), LPS-induced CXC (LIX), monocyte chemoattractant protein (MCP-1), macrophage colony-stimulating factor (M-CSF), or monokine induced by gamma interferon (MIG) (Additional file 2: Figure S1).

### Exposure to inhaled DEP and a high-fat diet results in altered cardiovascular disease biomarkers in cardiac tissue in male C57Bl/6 mice

**Fig. 7:**
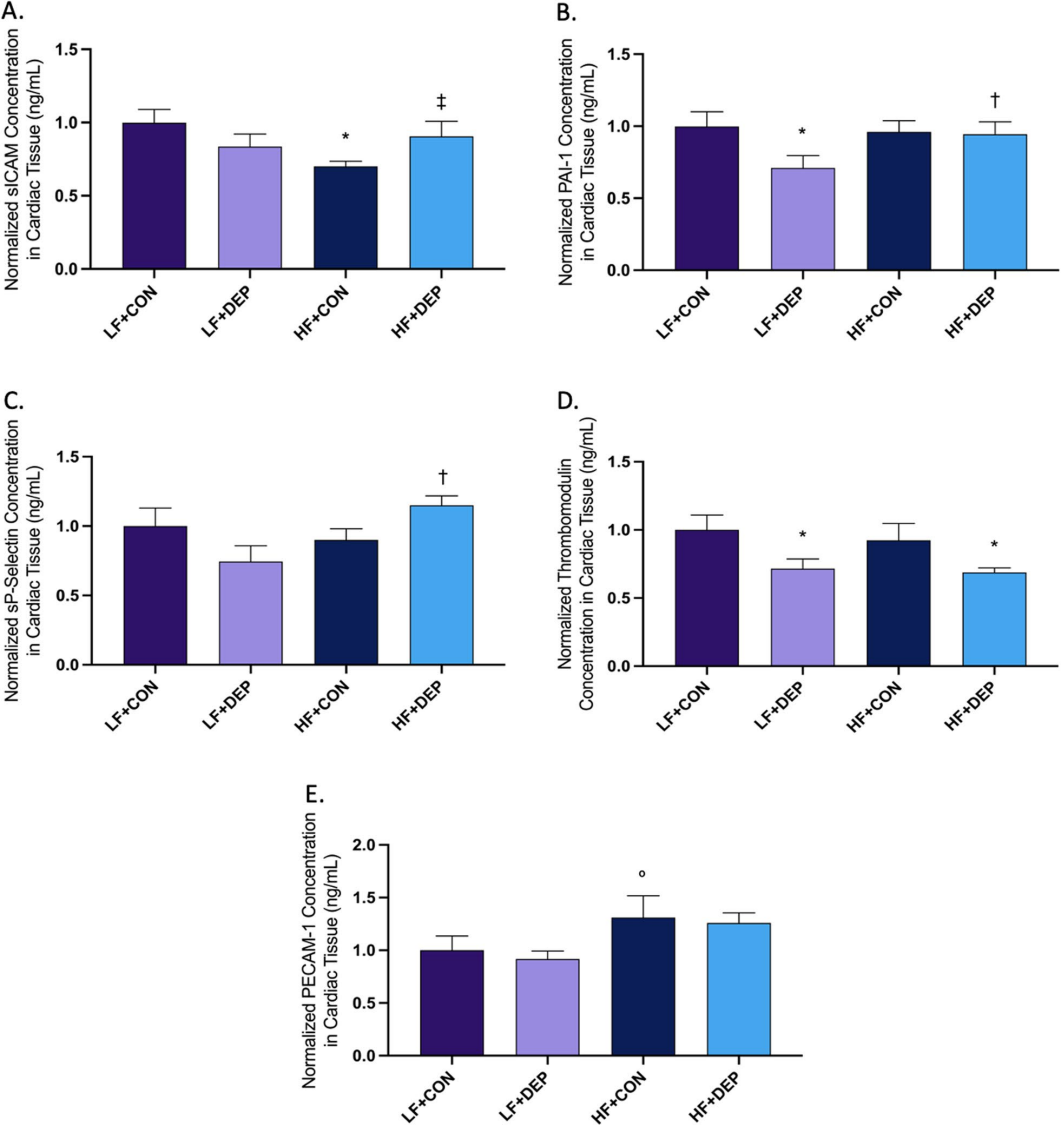
Exposure to inhaled DEP alters cardiovascular disease biomarkers. Mean normalized cardiovascular disease biomarkers: **A** siCAM, **B** PAI-1, **C** sP-Selectin, **D** thrombomodulin, and **E** PeCAM measured in cardiac tissue in C57Bl/6 male mice exposed to inhaled diesel exhaust particles (DEP- 35 μg PM) or saline control (CON) twice a week for four weeks on either low-fat (LF) or high-fat (HF) diet. Data are depicted as ± SEM normalized to total protein concentration with fold change with *p < 0.05 compared to LF + CON, †*p* < 0.05 compared to LF + DEP, ‡*p* < 0.05 compared to HF + CON, ◦*p* < 0.07 compared to LF + DEP by two-way ANOVA

As systemic inflammation is associated with the pathogenesis of CVD, we next investigated whether inhaled DEP exposure altered CVD biomarkers in cardiac tissue. We observed alterations in sICAM concentration with both DEP exposure and HF diet (Fig. 7A). When compared to LF + CON, there was a significant decrease in sICAM concentration in HF + CON (*p* = 0.010) group. Additionally, there was a significant increase in sICAM concentration in HF + DEP (*p* = 0.039) compared to HF + CON group. The respective F values for sICAM are as follows: exposure F = 0.058, diet F = 2.800, and exposure x diet F = 5.459. We also observed alterations in PAI-1 with exposure in respect to diet (Fig. 7B). Compared to LF + CON, there was a significant decrease in PAI-1 in the LF + DEP group (*p* = 0.043). There was also an increase in PAI-1 in HF + DEP group when compared to LF + DEP group (*p* = 0.033). The respective F values for PAI-1 are as follows: exposure F = 1.72, diet F = 1.854, and exposure x diet F = 3.123. sP-selectin concentration was statistically increased with inhaled DEP exposure and HF diet (Fig. 7C). When compared to LF + DEP, we observed a significant increase in sP-selectin concentration in HF + DEP (*p* = 0.010) group. The respective F values for sP-Selectin are as follows: exposure F = 0.001, diet F = 2.205, and exposure x diet F = 6.040. We found a statistical decrease in thrombomodulin concentration with DEP exposure (Fig. 7D). When compared to LF + CON, we observed a significant decrease in thrombomodulin for LF + DEP (*p* = 0.043) and HF + DEP (*p* = 0.028) groups. However, there was no statistical difference in HF + DEP (*p* = 0.088) group when compared to HF + CON group. The respective F values for thrombomodulin are as follows: exposure F = 8.076, diet F = 0.328, and exposure x diet F = 0.073. Lastly, we observed an increase in PECAM with HF diet (Fig. 7E), but no alterations were observed with DEP exposure. The respective F values for PECAM are as follows: exposure F = 0.269, diet F = 6.316, and exposure x diet F = 0.013.

### Exposure to inhaled DEP alters gut microbial profiles in mice on high-fat diet, which is mitigated by probiotic treatment

**Fig. 8:**
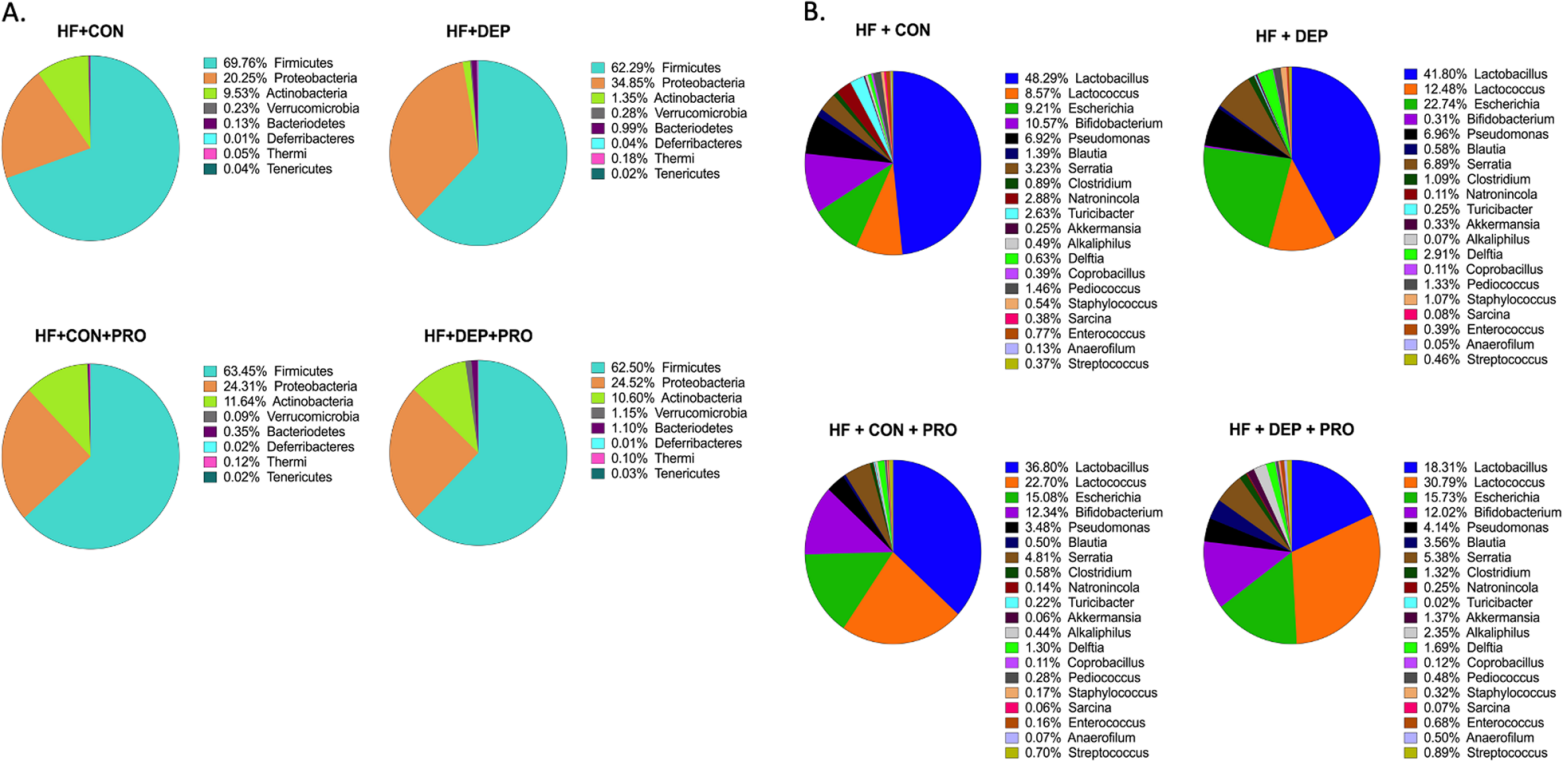
Probiotics attenuate DEP-driven expansion of Proteobacteria and decrease in Actinobacteria. Data shows normalized 100% relative abundance for **A** upper eight most abundant phyla pie chart with percentages and **B** upper 20 most abundant genus pie chart with percentages within the small intestine in C57Bl/6 male mice on high-fat (HF) diet exposed to diesel exhaust particles (DEP-35 μg PM) or saline control (CON) twice a week for 4 weeks with a subset of mice given 0.3 g/day (~ 7.5 × 10^7^ cfu/day) of Ecologic® Barrier probiotics (PRO) in the drinking water throughout the exposures. Top phyla and genera relative abundances were determined and analyzed in regards to respective control *n* = 6–7

**Fig. 9:**
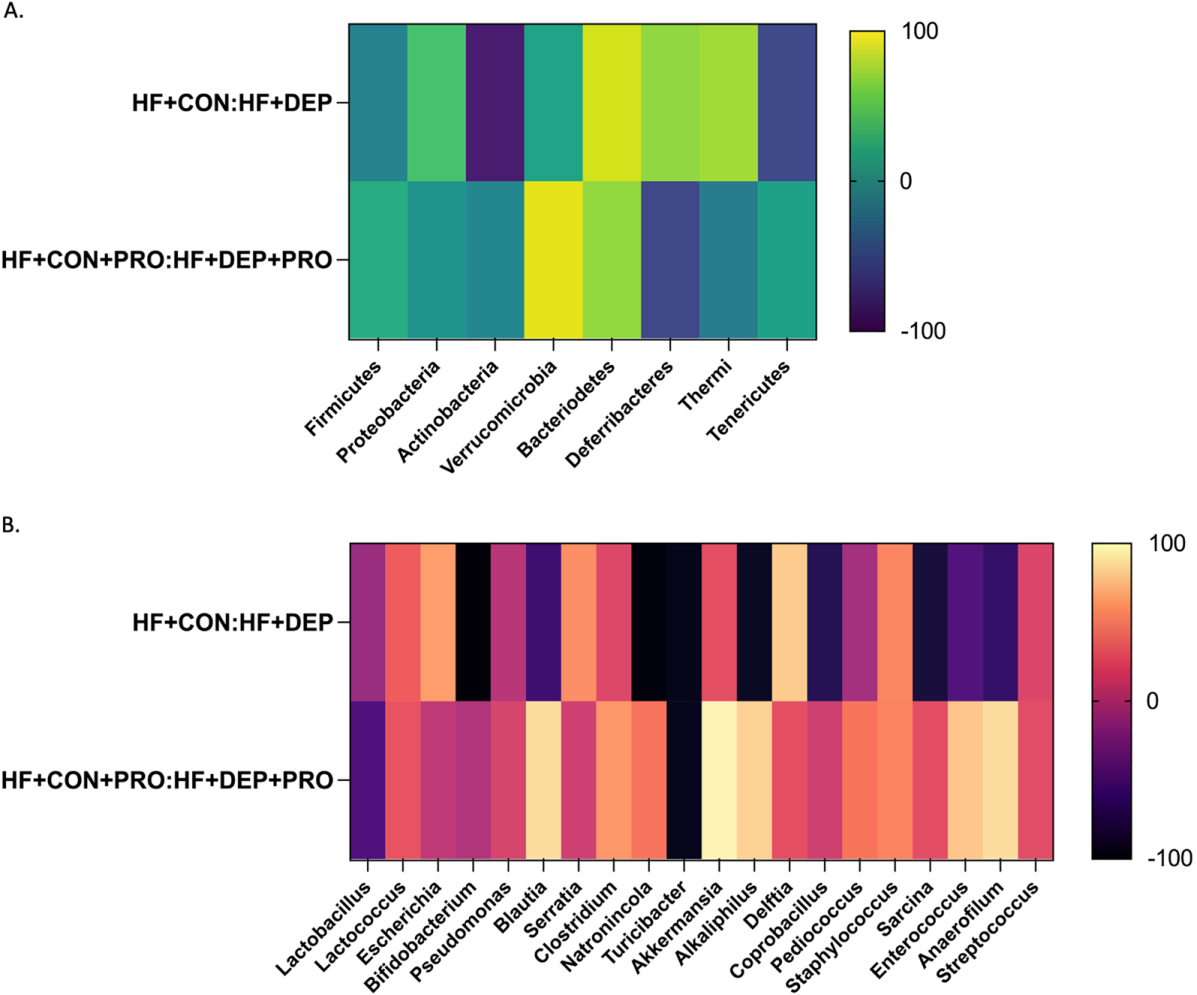
Exposure to inhaled DEP results in changes in microbial profiles which are mitigated in probiotic treated mice. Heat map shows the percent change in DEP or DEP + PRO compared to respective controls for most abundant phyla (**A**) and genus (**B**) in C57Bl/6 male mice on high-fat (HF) diet exposed to diesel exhaust particles (DEP- 35 μg PM) or saline control (CON) twice a week for four weeks with a subset of mice given 0.3 g/day (~ 7.5 × 10^7^ cfu/day) of Ecologic® Barrier probiotics (PRO) in the drinking water throughout the exposures. An increase in bacteria with DEP exposure is indicated by a positive percentage (yellow) and a decrease in bacteria with DEP exposure is indicated by a negative percentage (black). Values can be viewed in Table 3

To determine if probiotic-treatment can mitigate DEP mediated alterations in gut microbiome profiles of mice on HF diet, we treated a subset of mice with 0.3 g/day (~ 7.5 × 10^8^ cfu/day) of Ecologic® Barrier probiotics (PRO) in the drinking water throughout the exposures and determined the resulting microbial profiles. Figure 8 shows the relative abundance and percentage out of 100% of the top eight (A) phyla or top twenty (B) genus present in the small intestine of mice from each group. We found that probiotic treatment resulted in less change in Firmicutes with DEP exposure (Fig. 9). Probiotic treatment resulted in a 1.5% decrease in Firmicutes when comparing HF + DEP + PRO to HF + CON + PRO, which reduced the changes we observed between the HF + DEP and HF + CON groups by 9.2%. There was a 41.9% increase in the Proteobacteria in HF + DEP when compared to HF + CON. Alternatively, with probiotic treatment there was only a 0.85% increase in Proteobacteria in HF + DEP + PRO when compared to HF + CON + PRO. Thus, probiotic treatment attenuated the expansion of Proteobacteria in the gut of DEP-exposed animals on a HF diet. Within Proteobacteria, we found a 59.48% increase in Escherichia in HF + DEP when compared to HF + CON, however, that was reduced to only a 4.13% increase in Escherichia with probiotic treatment in HF + DEP + PRO when compared to HF + CON + PRO group. We also observed an 85.81% decrease in Actinobacteria and a 97.07% decrease in Bifidobacterium in HF + DEP compared to HF + CON. With probiotic treatment, we observed only an 8.9% decrease of Actinobacteria and a 2.2% decrease in Bifidobacterium in HF + DEP + PRO when compared to HF + CON + PRO. Thus, probiotic treatment diminished the overall reduction associated with DEP exposure of Actinobacteria by 79.61%. Additionally, we found an expansion of Verrucomicrobia with DEP exposure within probiotic-treated mice. Table 3 shows the percent change in relative abundance and difference in average percent relative abundance for HF + DEP to HF + CON and HF + DEP + PRO to HF + CON + PRO, which is represented in Fig. 9. The genera have been categorized into their respective phyla.

**Table 3:** Percent change in most abundant phyla and genus across exposure and probiotic-treatment groups

	HF + DEP compared to HF + CON		HF + DEP + PRO compared to HF + CON + PRO	
	Percent change (%)	Difference	Percent change (%)	Difference
*P_Firmicutes*	− 10.70	− 7.47	− 1.50	− 0.95
G_Lactobacillus	− 13.44	− 6.36	− 50.24	− 18.49
G_Lactococcus	+ 31.36	+ 3.93	+ 26.27	+ 8.18
G_Blautia	− 58.53	− 0.81	+ 86.00	+ 3.08
G_Clostridium	+ 18.78	+ 0.21	+ 56.47	+ 0.75
G_Natronincola	− 96.02	− 2.75	+ 41.67	+ 0.10
G_Turicibacter	− 90.67	− 2.37	− 90.10	− 0.20
G_Alkalphilius	− 86.48	− 0.42	+ 81.13	+ 1.92
G_Coprobacillus	− 70.62	− 0.27	+ 11.49	+ 0.01
G_Pediococcus	− 8.84	− 0.13	+ 42.21	+ 0.20
G_Staphylococcus	+ 49.13	+ .0.52	+ 42.21	+ 0.15
G_Sarcina	− 78.37	− 0.30	+ 22.67	+ 0.02
G_Enterococcus	− 49.26	− 0.38	+ 76.12	+ 0.15
G_Anaerofilum	− 63.14	− 0.08	+ 86.07	+ 0.43
G_Streptococcus	+ 18.16	− 0.82	+ 21.61	+ 0.19
*P_Proteobacteria*	+ 41.90	+ 14.6	+ 0.85	+ 0.21
G_Escherichia	+ 59.48	+ 13.53	+ 4.13	+ 0.65
G_Pseudomonas	+ 0.52	+ 0.04	+ 15.79	+ 0.65
G_Serratia	+ 53.06	+ 3.66	+ 10.70	+ 0.58
G_Delftia	+ 78.43	+ 2.28	+ 23.28	+ 0.41
*P_Actinobacteria*	− 85.81	− 8.18	− 8.91	− 1.04
G_Bifidobacterium	− 97.07	− 10.26	− 2.62	− 0.32
*P_Verrucomicrobia*	+ 16.06	+ 0.04	+ 92.47	+ 1.06
G_Akkermansia	+ 23.20	+ 0.08	+ 95.37	+ 1.30
P_Bacteriodetes	+ 86.91	+ 0.86	+ 67.85	+ 0.75
P_Deferribacteres	+ 67.23	+ 0.03	− 57.18	− 0.01
P_Thermi	+ 72.50	+ 0.13	− 16.88	− 0.01
P_Tenericutes	− 55.75	− 0.02	+ 13.10	+ 0.01

DEP, diesel exhaust particle; CON, saline controls; HF, high-fat diet; PRO, probiotic treatment

**Fig. 10:**
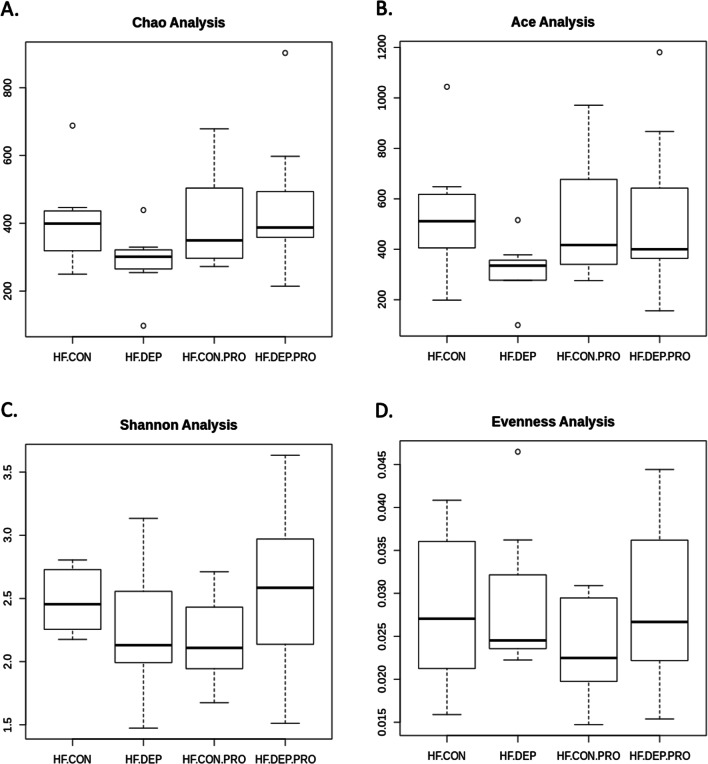
Bacterial alpha diversity analysis between exposure and probiotic treatment. Alpha diversity analysis using **A** Chao, **B** Ace, **C** Shannon, and **D** Evenness compared in C57Bl/6 male mice on high-fat (HF) diet exposed to diesel exhaust particles (DEP- 35 μg PM) or saline control (CON) twice a week for four weeks with a subset of mice given 0.3 g/day (~ 7.5 × 10^7^ cfu/day) of Ecologic® Barrier probiotics (PRO) in the drinking water throughout the exposures. *n* = 6–7

**Fig. 11:**
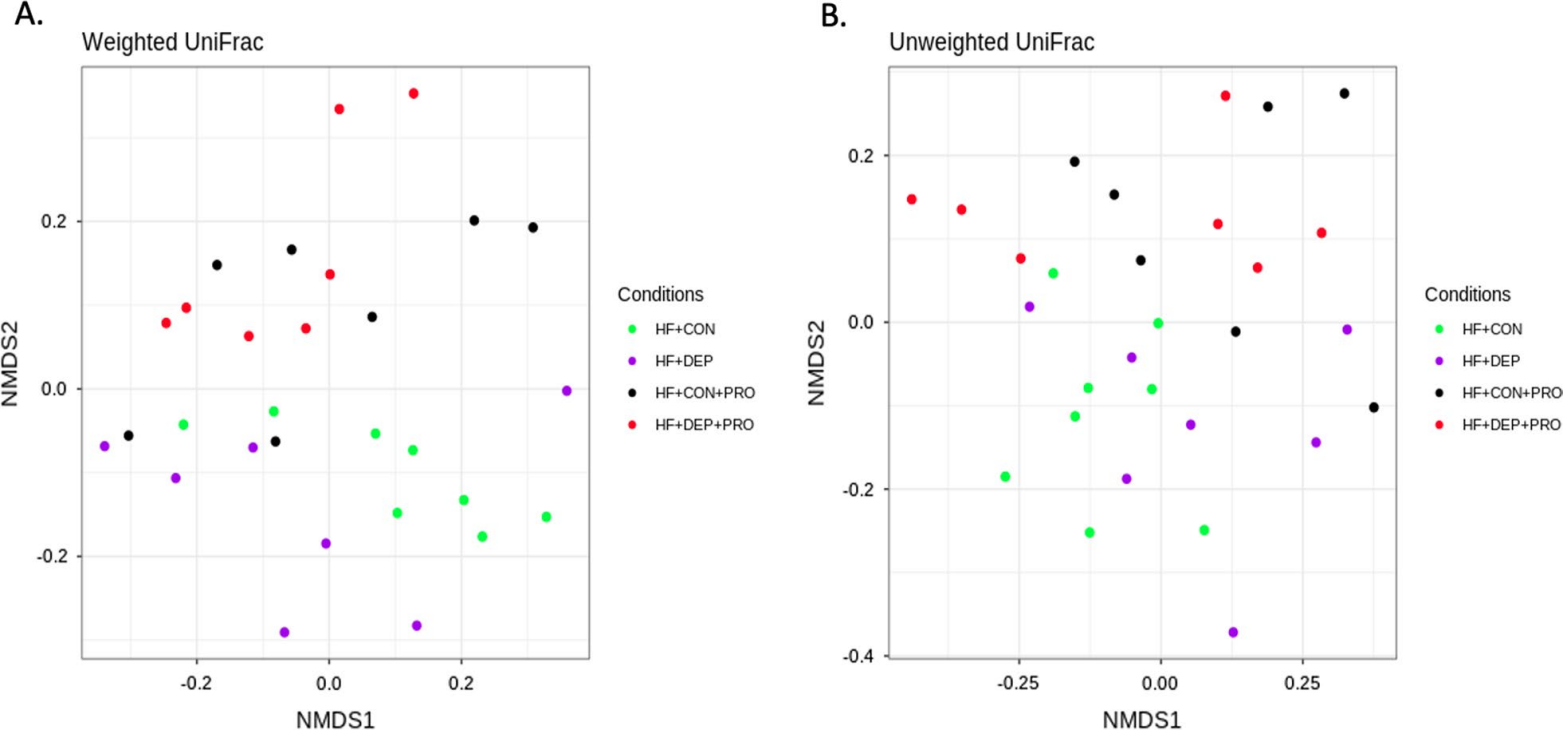
Bacterial beta diversity analysis between exposure and probiotic treatment. Beta diversity was calculated using UniFrac distances and Principle Coordinates Analysis to show the similarities in microbial communities in both **A** weighted and **B** unweighted analyses. Each dot represents compared in C57Bl/6 male mice on high-fat (HF) diet exposed to diesel exhaust particles (DEP- 35 μg PM) or saline control (CON) twice a week for four weeks with a subset of mice given 0.3 g/day (~ 7.5 × 10^7^ cfu/day) of Ecologic® Barrier probiotics (PRO) in the drinking water throughout the exposures. *n* = 6–7

The α-diversity of microbial profiles in the small intestine was assessed by richness (Chao and ACE) and diversity (Shannon and Evenness) (Fig. 10). A decrease in diversity and richness were observed with DEP exposure between HF + CON and HF + DEP. Coversely, with probiotic treatment, we observe an increase in diversity and richness in both the HF + CON + PRO and HF + DEP + PRO groups. There were no statistical differences in alpha diversity with exposure, probiotic treatment, or interaction by ANOVA. However, there were statistical differences in β-diversity in microbial profiles within the small intestine between these four groups. Figure 11 shows the (A) weighted PCoA and (B) unweighted PCoA plots based on UniFrac distance using the fraction of total variance of bacteria represented by each axis with each dot representing one animal within each group. Both weighted and unweighted PCoA plots show dispersing of the groups with respect to probiotic treatment, but not DEP exposure. We performed Analysis of Molecular Variance (AMOVA) to assess the variations among different groups. AMOVA showed UniFrac distances with significant differences for comparisons between all four groups (HF + CON:HF + CON + PRO:HF + DEP:HF + DEP + PRO) with *p*-value of 0.002, between HF + CON and HF + CON + PRO groups with p-value of 0.001, between HF + CON and HF + DEP + PRO groups with *p*-value of 0.003, between HF + DEP and HF + DEP + PRO groups with *p*-value of 0.032 (Table 4).

**Table 4:** AMOVA analysis of microbial profiles from small intestine

Exposure Groups	SS	Df	MS	Fs	*p*-value
*HF* + *CON- * versus* HF* + *CON* + *PRO * versus* HF* + *DEP * versus* HF* + *DEP* + *PRO*				3.57114	0.002
Among	2.08605	3	0.69535		
Within	4.86785	25	0.19471		
Total	6.9539	28			
*HF* + *CON * versus* HF* + *CON* + *PRO*				7.80237	0.001
Among	1.37017	1	1.37017		
Within	2.28292	13	0.17561		
Total	3.65309	14			
*HF* + *CON * versus* HF* + *DEP*				1.14753	0.364
Among	0.26276	1	0.262757		
Within	2.97668	13	0.228976		
Total	3.23944	14			
*HF* + *CON * versus* HF* + *DEP* + *PRO*				4.92282	0.003
Among	0.98308	1	0.983079		
Within	2.59608	13	0.199698		
Total	3.57916	14			
*HF* + *CON* + *PRO * versus* HF* + *DEP*				4.02749	0.006
Among	0.76246	1	0.762461		
Within	2.27177	12	0.189314		
Total	3.03423	13			
*HF* + *CON* + *PRO * versus* HF* + *DEP* + *PRO*				1.30782	0.265
Among	0.20611	1	0.206109		
Within	1.89117	12	0.157597		
Total	2.09728	13			
*HF* + *DEP * versus* HF* + *DEP* + *PRO*				2.55175	0.032
Among	0.549675	1	0.549675		
Within	2.58493	12	0.215411		
Total	3.13461	13			

DEP, diesel exhaust particles; CON, saline controls; HF, high-fat diet; LF, low-fat diet; SS, sum of squares; Df; degrees of freedom; MS, mean of squares

### Probiotic strains are established in the small intestine of mice exposed to inhaled DEP and control

**Fig. 12:**
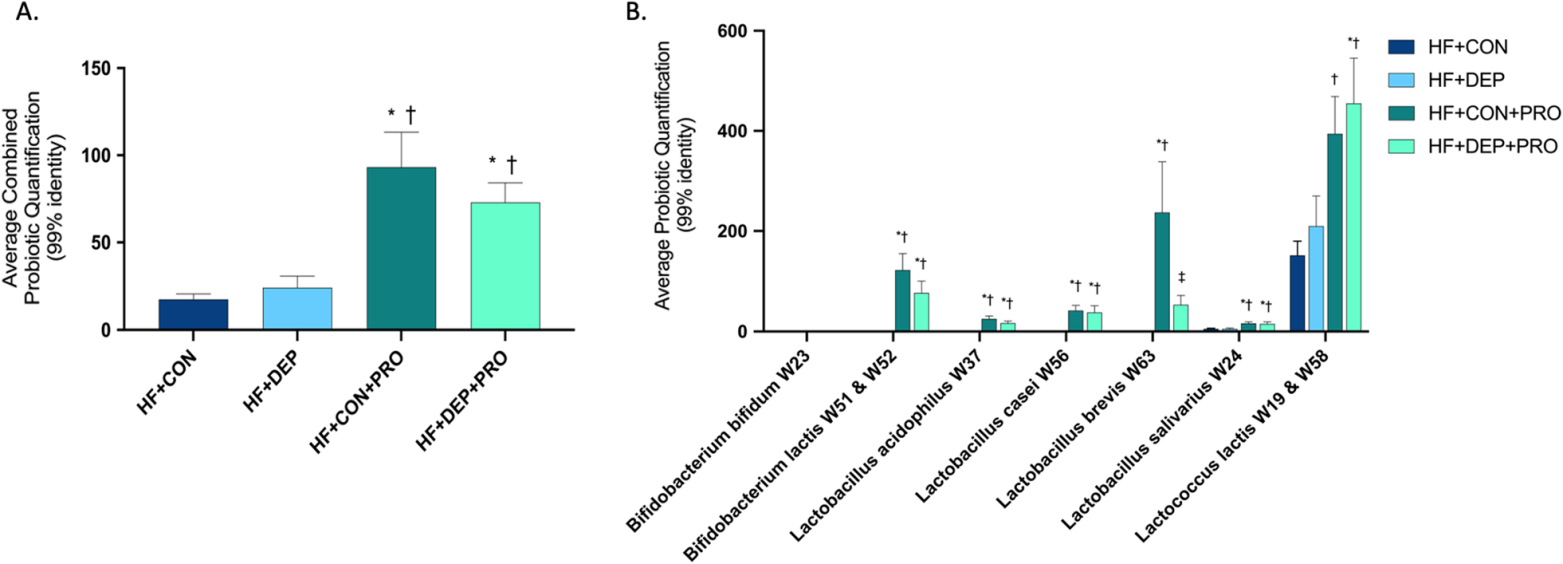
Abundance of probiotic bacterial strains within the small intestine. Quantification of individual probiotic strains (**A**) and average combined probiotic (**B**) in C57Bl/6 male mice on high-fat (HF) diet exposed to diesel exhaust particles (DEP- 35 μg PM) or saline control (CON) twice a week for four weeks with a subset of mice given 0.3 g/day (~ 7.5 × 10^7^ cfu/day) of Ecologic® Barrier probiotics (PRO) in the drinking water throughout the exposures. Quantification of probiotic strains was performed using sequences clustered into operational taxonomic units (OTUs) based on 99% similarity identity, then the V4 region 16S rRNA gene bacterial samples were aligned as a query via global alignment against a database constructed of the 16S rRNA sequences for the probiotics used in the study. Data are depicted as ± SEM with *p < 0.05 compared to HF + CON, †*p* < 0.05 compared to HF + DEP, and ‡*p* < 0.05 compared to HF + CON + DEP by two-way ANOVA

We next quantified the probiotic strains to confirm their presence within the small intestine (Fig. 12). We observed significantly increased levels of bacterial strains present in the probiotics supplement in the small intestine of mice in the probiotic treatment group, compared to those in the control group. Figure 12B shows the individual strains’ presence in the small intestine for each of the four groups. Two *Bifidobacterium lactis* strains (W51 and W52) and two *Lactococcus lactis* strains (W19 and W58) were combined due to > 99% identity similarity between the two strains.

### Probiotic treatment decreases LPS levels in mice on high-fat diet exposed to inhaled DEP

**Fig. 13:**
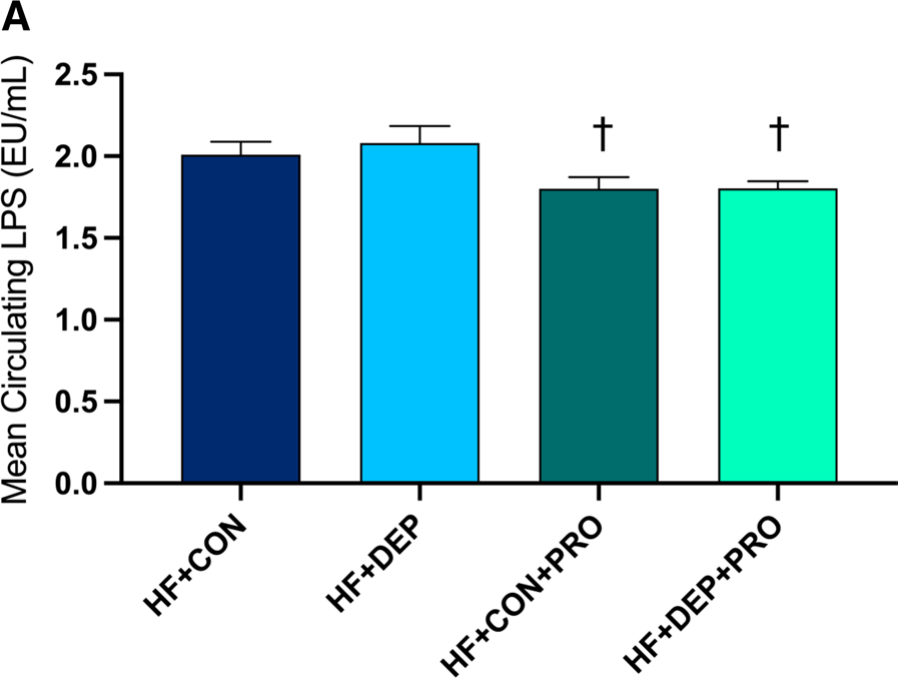
Probiotic treatment results in a reduction of circulating LPS. Mean circulating (**A**) LPS in C57Bl/6 male mice on high-fat (HF) diet exposed to diesel exhaust particles (DEP- 35 μg PM) or saline control (CON) twice a week for four weeks with a subset of mice given 0.3 g/day (~ 7.5 × 10^7^ cfu/day) of Ecologic® Barrier probiotics (PRO) in the drinking water throughout the exposures. Data are depicted as ± SEM with *p < 0.05 compared to HF + CON and †*p* < 0.05 compared to HF + DEP by two-way ANOVA

To determine whether probiotic-treatment attenuated either DEP or HF diet mediated increases in LPS, we assessed circulating LPS levels (Fig. 13). Probiotic-treatment significantly decreased circulating LPS in mice on a HF diet (*p* = 0.025) and those on a HF diet exposed to DEP (*p* = 0.026), compared to the HF + DEP group. The respective F values for circulating LPS are as follows: exposure F = 0.202, probiotics F = 8.769, and exposure x probiotics F = 0.171.

### Probiotic treatment alters circulating systemic inflammatory biomarkers in C57Bl/6 mice exposed to inhaled DEP on a high-fat diet

**Fig. 14:**
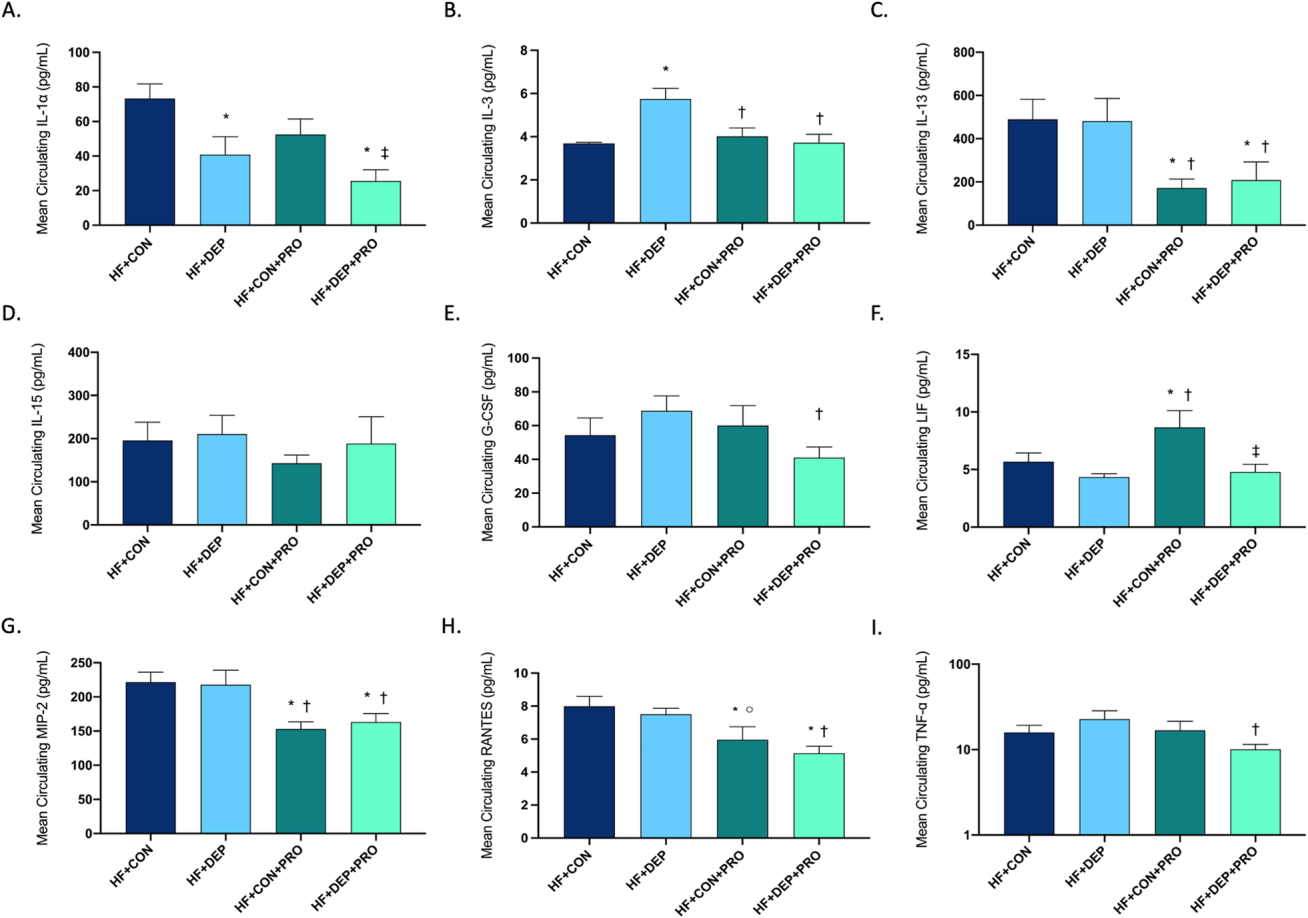
Probiotic treatment alters circulating cytokine concentrations in mice on high-fat diet exposed to inhaled DEP. Mean concentration for circulating inflammatory factors: **A** IL-1α, **B** IL-3, **C** IL-13, **D** IL-15, **E** G-CSF, **F** LIF, **G** MIP-2, **H** RANTES, and **I** TNF-α measured in plasma in C57Bl/6 male mice on high-fat (HF) diet exposed to diesel exhaust particles (DEP- 35 μg PM) or saline control (CON) twice a week for four weeks with a subset of mice given 0.3 g/day (~ 7.5 × 10^7^ cfu/day) of Ecologic® Barrier probiotics (PRO) in the drinking water throughout the exposures. Data are depicted as ± SEM with *p < 0.05 compared to HF + CON, †*p* < 0.05 compared to HF + DEP, and ‡*p* < 0.05 compared to HF + CON + PRO, and ◦*p* < 0.07 compared to HF + DEP by two-way ANOVA

Probiotics have previously been shown to decrease ILs, both locally and systemically [37], thus we measured circulating IL concentrations in response to DEP exposure in HF diet mice ± probiotics (Fig. 14). We observed a significant decrease in IL-1α with exposure and probiotic treatment (Fig. 14A). Compared to HF + CON, there is a statistical decrease in IL-1α for HF + DEP (*p* = 0.014) and HF + DEP + PRO (*p* < 0.001) groups. Additionally, there was a statistical decrease in HF + DEP + PRO (*p* = 0.033) when compared to HF + CON + PRO. The respective F values for IL-1α are as follows: exposure F = 12.27, probiotics F = 4.548, and exposure x probiotics F = 0.112. Conversely, there was a statistical increase in IL-3 with DEP exposure and probiotics (Fig. 14B). When compared to HF + CON, there is a statistical increase in IL-3 in HF + DEP (*p* = 0.003). Compared to HF + DEP, there is a statistical decrease in IL-3 for both HF + CON + PRO (*p* = 0.009) and HF + DEP + PRO (*p* = 0.006) groups. The respective F values for IL-3 are as follows: exposure F = 3.984, probiotics F = 3.737, and exposure x probiotics F = 7.182. There was an overall reduction of IL-13 noted with probiotic treatment (Fig. 14C). When compared to HF + CON, there is a significant decrease in IL-13 for HF + CON + PRO (*p* = 0.018) and HF + DEP + PRO (*p* = 0.033) groups. Furthermore, compared to HF + DEP, there was a significant decrease in IL-13 for HF + CON + PRO (*p* = 0.024) and HF + DEP + PRO (*p* = 0.044) groups. The respective F values for IL-13 are as follows: exposure F = 0.024, probiotics F = 10.99, and exposure x treatment F = 0.063. We did not observe any statistical differences for IL-15 in the HF ± probiotics groups (Fig. 14D).

Probiotic-treatment has been reported to mediate anti-inflammatory effects [37], thus we analyzed the outcome of probiotic treatment in our study on systemic cytokine and chemokine concentration in our study animals (Fig. 14). When compared to HF + DEP, there is a significant decrease in G-CSF in HF + DEP + PRO group (Fig. 14E, *p* = 0.048). The respective F values for G-CSF are as follows: exposure F = 0.057, probiotics F = 1.291, and exposure x probiotics F = 3.021. Interestingly, probiotic treatment was associated with a significant increase in circulating LIF for HF + CON + PRO when compared to HF + CON (Fig. 14F; *p* = 0.040) and HF + DEP (*p* = 0.020). Additionally, there was a significant decrease in LIF in HF + DEP + PRO (*p* = 0.034) when compared to HF + CON + PRO. The respective F values for LIF are as follows: exposure F = 5.309, probiotics F = 2.296, and exposure x probiotics F = 1.258. We observed a significant reduction in MIP-2 with probiotics (Fig. 14G). When compared to HF + CON and HF + DEP, there is a significant decrease in HF + CON + PRO (*p* = 0.005 and *p* = 0.010, respectively) and HF + DEP + PRO (*p* = 0.008 and *p* = 0.017, respectively) groups. The respective F values for MIP-2 are as follows: exposure F = 0.047, probiotics F = 16.93, and exposure x probiotics F = 0.209. Probiotic-treatment also mediated a reduction in RANTES in our study animals (Fig. 14H). Similarly, when compared to HF + CON, we see a significant decrease in RANTES for HF + CON + PRO (*p* = 0.020) and HF + DEP + PRO (*p* = 0.003) groups. There was also a decrease in RANTES in HF + CON + PRO (*p* = 0.069) and HF + DEP + PRO (*p* = 0.011) when compared to HF + DEP. The respective F values for RANTES are as follows: exposure F = 1.291, probiotics F = 14.39, and exposure x probiotics F = 0.081. Lastly, we observed a significant decrease in TNF-α with probiotic treatment in the HF + DEP + PRO (*p* = 0.028) group when compared to HF + DEP group (Fig. 14I). The respective F values for TNF-α are as follows: exposure F < 0.001, probiotics F = 2.242*,* and exposure x probiotics F = 3.047*.*

There were no statistical differences for IFNγ, IL-2, IL-9, IL-12(p20), LIX, MCP-1, M-CSF, and MIG observed across any of the DEP exposure or treatment groups (Additional file 3: Figure S2). We did note a statistical decrease in IL-1β when comparing HF + CON + PRO to HF + CON (*p* = 0.041) and HF + DEP (*p* = 0.005) using an unpaired t-test (Additional file 3: Figure S2); however, could not perform a two-way ANOVA due to the HF + DEP + PRO group IL-1β measurements being below the standard curve.

### Probiotic treatment mitigates cardiovascular stress response in C57Bl/6 mice exposed to inhaled DEP on high-fat diet

**Fig. 15:**
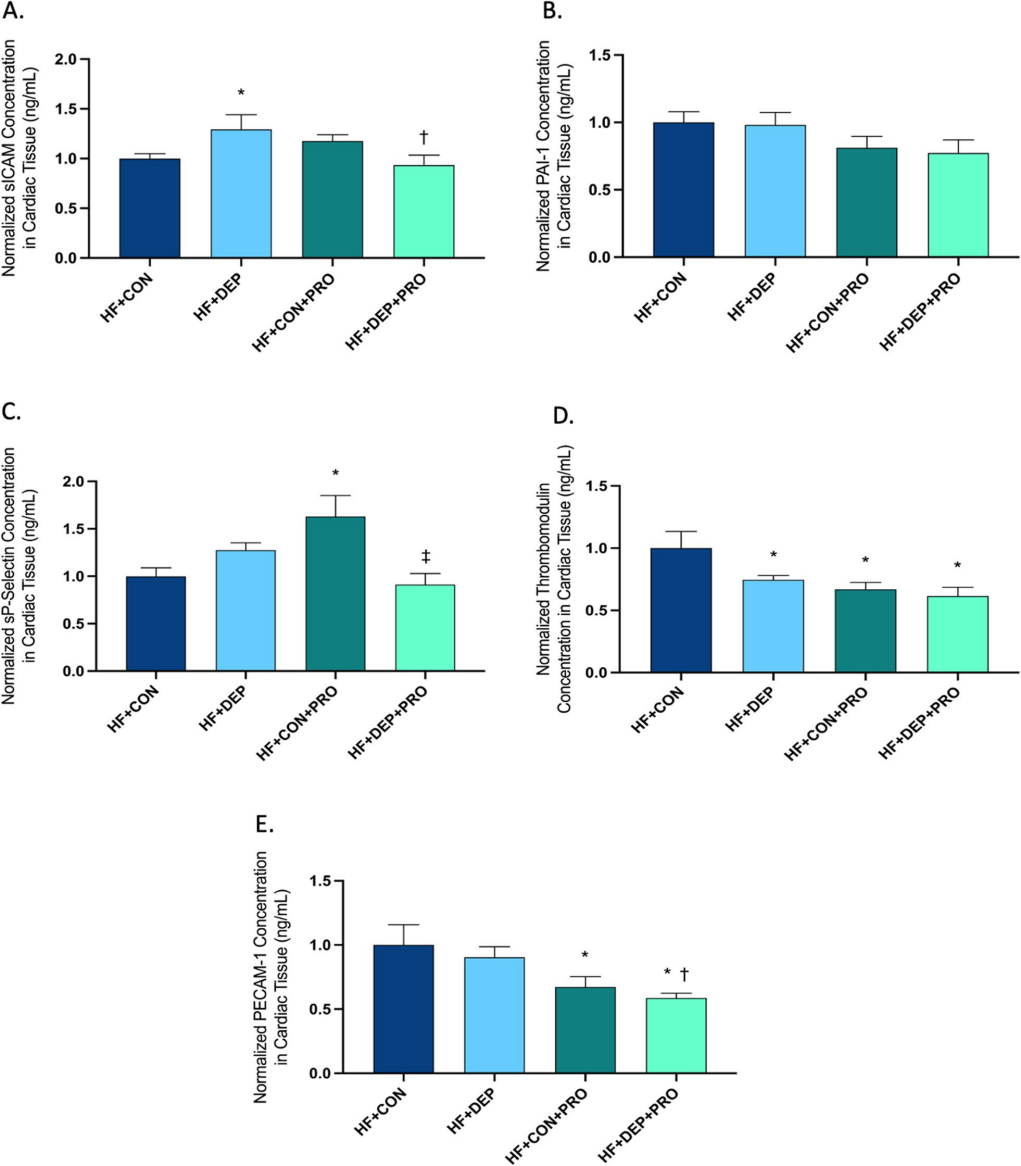
Probiotic treatment alters cardiovascular biomarkers in mice exposed to inhaled DEP on a high-fat diet. Mean normalized cardiovascular disease biomarkers: **A** sICAM, **B** PAI-1, **C** sP-Selectin, **D** thrombomodulin, and **E** PeCAM measured in cardiac tissue C57Bl/6 male mice on high-fat (HF) diet exposed to diesel exhaust particles (DEP- 35 μg PM) or saline control (CON) twice a week for four weeks with a subset of mice given 0.3 g/day (~ 7.5 × 10^7^ cfu/day) of Ecologic® Barrier probiotics (PRO) in the drinking water throughout the DEP exposures. Data are depicted as ± SEM normalized to total protein concentration with fold change with *p < 0.05 compared to HF + CON, †*p* < 0.05 compared to HF + DEP, and ‡*p* < 0.05 compared to HF + CON + PRO by two-way ANOVA

To determine whether probiotic treatment altered CVD biomarkers in our study animals, we analyzed cardiac tissue. Probiotic-treatment normalized increased sICAM levels observed in HF + DEP animals (Fig. 15A; *p* = 0.018). The respective F values for sICAM are as follows: exposure F = 0.078, probiotics F = 0.874, and exposure x probiotics F = 7.505. There were no statistical differences for PAI-1 observed across any groups (Fig. 15B). However, probiotic treatment resulted in altered circulating sP-selectin levels in both CON and DEP exposed animals (Fig. 15C). When compared to HF + CON, there is a significant increase in sP-selectin in HF + CON + PRO (*p* = 0.012). Furthermore, sP-selectin is decreased in HF + DEP + PRO (*p* = 0.002) compared to HF + CON + PRO. We do not observe the same trend when comparing HF + CON to HF + DEP (*p* = 0.233). Additionally, there are no differences noted between HF + DEP and HF + DEP + PRO groups (*p* = 0.085). The respective F values for sP-selectin are as follows: exposure F = 2.164, probiotics F = 0.780, and exposure x probiotics F = 10.96. We observed a significant decrease in thrombomodulin with probiotic treatment (Fig. 15D). When compared to HF + CON, there is a significant decrease in thrombomodulin with HF + DEP (*p* = 0.047), HF + CON + PRO (*p* = 0.018), and HF + DEP + PRO (*p* = 0.005). Additionally, there was no significant difference between HF + CON + PRO and HF + DEP + PRO (*p* = 0.675). The respective F values for thrombomodulin are as follows: exposure F = 3.214, probiotics F = 7.184, and exposure x probiotics F = 1.37. Lastly, probiotic treatment resulted in a significant decrease in circulating PeCAM (Fig. 15E). When compared to HF + CON, there is a significant decrease in PeCAM for HF + CON + PRO (*p* = 0.022) and HF + DEP + PRO (*p* = 0.005). Additionally, there was a significant decrease in PeCAM in HF + DEP + PRO (*p* = 0.014) when compared to HF + DEP. The respective F values for PeCAM are as follows: exposure F = 1.058, probiotics F = 13.63, and exposure x probiotics F = 0.004.

## Discussion

**Fig. 16:**
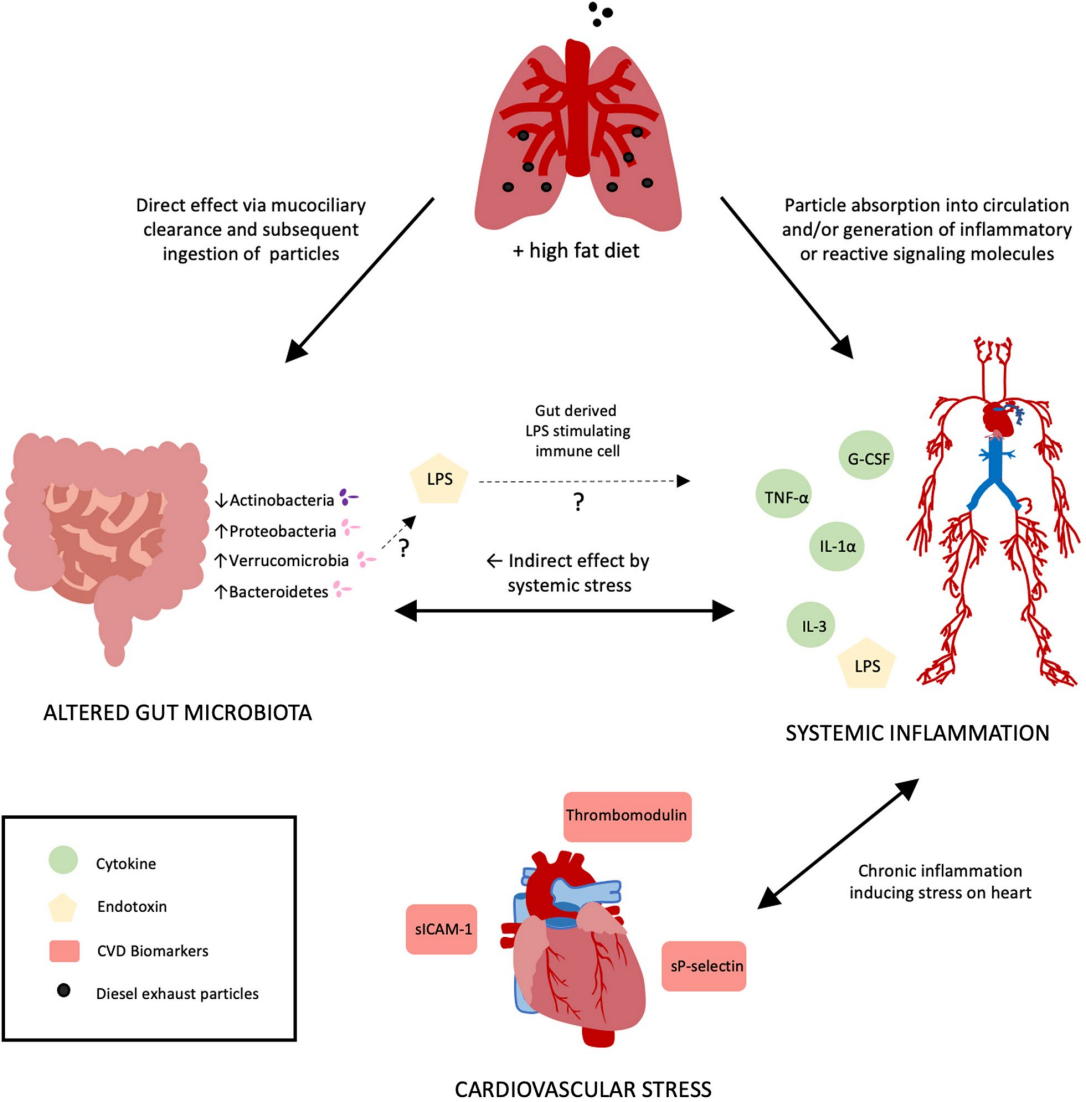
Proposed mechanism of inhaled DEP and HF diet on the gut microbiome, systemic inflammation, and CVD biomarkers. DEP from the lungs can affect the gut by indirect effects by absorption through the lungs into circulation or by direct effect via mucociliary clearance and subsequent ingestion and entry into the gastrointestinal tract. Systemic responses increase inflammation and cytokine signaling via granulocyte colony-stimulating factor (G-CSF), tumor necrosis factor alpha (TNF-α), interleukin (IL)-1α, and IL-3, which can stress the gut causing alterations in the gut microbiome. Alterations in the gut microbiome associated with inhaled DEP exposure include decreased Actinobacteria and expansion of Proteobacteria, Verrucomicrobia, and Bacteroidetes, which could likely be the source of lipopolysaccharide (LPS) infiltration into circulation further exacerbating inflammatory cytokine concentration in circulation. The overall increase in DEP osure mediated systemic inflammation results in the early concentration of cardiovascular disease (CVD) biomarkers such as thrombomodulin, soluble intracellular adhesion molecule (sICAM), and soluble platelet selection (sP-selectin). This persistent change in microbial profiles and inflammation is a possible contributor to DEP-mediated CVD

Studies have shown a correlation between exposure to inhaled environmental air pollutants and systemic inflammation, CVD, and alterations in the gut microbiome [6, 9, 16, 17]; HF diet is also a known contributing factor to these disease states [19, 43–45]. However, few studies have investigated how the gut microbial composition changes correspond with systemic inflammation and markers of CVD resulting from inhaled traffic-generated PM exposure coupled with a HF diet. There are three potential mechanisms through which inhaled air pollution can alter the gut microbiome: (1) through a direct effect, where inhaled PM can be trapped in the mucociliary tract and subsequently expelled and swallowed, (2) through an indirect effect, where PM is inhaled into the lungs and are absorbed across the respiratory membrane into the systemic circulation acting via the gut vasculature, or (3) through an indirect effect, where inhaled PM promotes lung microbial dysbiosis, oxidative stress, or and inflammatory signaling factors that can influence the gut microbiome via the circulation (Fig. 16). We have previously reported that inhaled DEP exposure from these same study animals resulted in a significant expansion of Proteobacteria within the lungs, associated with inflammation and oxidative stress, which was attenuated through probiotic treatment [46]. Considering the exacerbated responses to DEP exposure in the HF diet animals, we decided to use a probiotic treatment in this study group to determine if we could mitigate the exposure and diet-mediated alterations in microbiota profiles and systemic responses.

The influence of the microbiome on health and homeostasis is profound, and there is a need to understand how environmental exposures alter the microbiome and its correlative responses of the host. We identified the major bacterial profiles in the small intestine of mice exposed to inhaled DEP on a LF and HF diet to understand further the complex relationship that air pollution PM exposures have on the gut microbiome within two different applicable dietary models. We further investigated the role of the gut microbiome in homeostasis by supplementing probiotics to maintain and support the microbial profiles and determine the microbiome’s influence on systemic responses.

Furthermore, we see an expansion of Proteobacteria in the intestines of HF groups with DEP exposure, which is not observed with the LF groups. *Escherichia*, a bacterium in the phyla Proteobacteria, has been shown to proliferate and favor a diet higher in fats [47]. Interestingly, we did not observe this alteration when we compared our LF + CON and HF + CON groups in the current study. This may be due to the sub-chronic time point of the study, age of animals, and/or lack of secondary physiological insult like that observed with the DEP-exposed animals. Proteobacteria are considered commensal, functional bacteria in the gut; however, its expansion can be associated with diseases and is considered a source of endotoxemia [48]. We propose that the HF diet provides an environment for Proteobacteria expansion, reducing the abundance of the gram-positive genera *Turicibacter,* observed to be decreased in the HF diet group. In probiotic-treated mice, we did not observe an expansion of Proteobacteria and *Escherichia* with DEP exposure; however, there was an expansion of *Lactococcus*. It is plausible that probiotic supplementation in HF + DEP + PRO group allows for *Lactococcus* and *Bifidobacterium* to colonize and antagonize *Escherichia* expansion we observe in HF + DEP group. There was an expansion of Verrucomicrobia and *Akkermansia* with DEP exposure in the LF, HF, and HF + PRO groups. *Akkermansia* has recently gained attention for its importance in mucin degradation in the gut; however, there are conflicting reports of its involvement in health and disease [49]. It is possible that the expansion of *Akkermansia* observed in our study animals may result from ingestion of DEP from mucociliary clearance, promoting gut dysbiosis. However, further investigation is needed to identify these proposed mechanisms.

The mice in this study exhibit an overall low abundance of the phyla Bacteroidetes; however, previous studies have shown that Firmicutes and Proteobacteria dominate the proximal portions of the small intestine [50]. It is also plausible that the study mice were colonized with a microbiome with a low abundance of Bacteroidetes from maternal transfer or the rearing/storage facility [51]. We observed an expansion in the phyla Bacteroidetes with DEP exposure across all groups. These findings agree with a previous study that observed similar changes in the small intestine and colon of mice exposed to inhaled ambient PM [9]. We also found the same expansion in our previous study in woodsmoke exposed ApoE^−/−^ mice [16].

When considering alpha diversity, we did not observe any significant differences in the species richness or diversity from exposure; however, alterations were noted in these endpoints in the probiotic-treated group (HF + DEP + PRO) compared HF + DEP group. When looking at beta diversity, our PCoA plots do not show clustering based on DEP exposure but instead show differences between LF and HF diet groups and HF and HF + PRO groups, which were confirmed with AMOVA. Thus, based on our results, the HF diet and probiotic treatment shift the beta diversity within the microbiome in the current study. It is plausible that we do not see changes in diversity with DEP exposure in this study due to our exposure method by OA of DEP directly into the airway. This method bypasses the oronasal region and thus results in fewer particles being ingested, except through mucociliary clearance.

Both DEP exposure and HF diet promoted a significant increase in circulating LPS levels. While it is well established that HF diet is associated with gut-derived endotoxemia, to our knowledge, no studies have shown that inhaled DEP increases circulating LPS [30, 36]. The phyla Proteobacteria is commonly considered one of the sources of gut-derived LPS, yet we observed no change in Proteobacteria within LF + DEP animals, which did show elevated LPS levels. However, there was an increase in two gram-negative genera within Proteobacteria phyla, *Pseudomonas,* and *Delftia*, in addition to Bacteroidetes, which could serve as a source of LPS. Importantly, the levels of LPS in the NIST DEP material used for these studies was previously measured by our laboratory and determined to be negligible; thus, it is not likely to be a contributing source of circulating LPS [46]. Probiotic treatment reduced systemic LPS levels in the HF + DEP group; however, there is no reduction of Proteobacteria compared to HF + CON. There is some evidence that Bifidobacterium and Lactobacillus can reduce LPS, which may account for the reduced LPS levels observed in the probiotic-treated groups [37]. It is plausible that the probiotic treatment mitigates translocation of microbial LPSs, possibly by preventing damage to the intestinal epithelial barrier caused by the DEP exposure and HF diet.

We observed a pronounced reduction in both the phyla Actinobacteria and related genera *Bifidobacterium* in response to inhaled DEP, regardless of diet. Actinobacteria are commensal bacteria that play a dynamic role in the microbiome by producing biologically active substances, such as bacteriocins, that provide antagonistic effects within the gut microbiome [52]. Our probiotics contain a propriety blend of 9 bacteria, including three strains of *Bifidobacterium*. We observed attenuation of Actinobacteria and *Bifidobacterium* with probiotic treatment in the DEP exposed and control group, suggesting that inhaled DEP with HF diet affects the colonization of *Bifidobacterium* and show that probiotic treatment can mitigate this response. Moreover, *Bifidobacterium* probiotics are known to modulate the immune system and reduce serum cytokines, proving a possible source of anti-inflammatory effects observed in this study [41].

Interleukins (IL) are a family of cytokines produced by cells that mediate inflammatory responses during tissue damage, infection, or other toxic insults. IL-1α is an inflammatory cytokine expressed by immune, epithelial, endothelial, and stromal cells and responds to various stimuli, including LPS [53]. Anti-IL-1α antibodies have been shown to reduce inflammation and promote the repair of the intestinal epithelial barrier in mice, suggesting a decrease in IL-1α can be protective [54]. Additionally, IL-1α^−/−^ mice have been reported to have a significant decrease in gut *Akkermansia muciniphila* bacteria and increased intestinal integrity following dextran sulfate sodium (DSS)-induced colitis compared to wildtype mice, suggesting that IL-1α can regulate colonization of *Akkermansia* in the gut [55]. *Akkermansia,* in the phyla Verrucomicrobia, has been shown to degrade intestinal mucin and alter intestinal lining permeability, which allows infiltration of LPS and other toxins into circulation [49, 55]. Alternatively, some studies have shown that *Akkermansia muciniphila* is reduced with inflammatory bowel disease and promotes a healthy mucin layer in the intestines [49]. Our study resulted in a significant decrease in circulating IL-1α, associated with increased phyla Verrucomicrobia and genus *Akkermansia,* with DEP exposure regardless of diet or probiotic treatment. We also measured circulating IL-1β, a potent pro-inflammatory cytokine that mediates tissue repair. Although not statistically significant, we observed an increase in IL-1β with DEP exposure in both LF and HF diet-fed animals (Additional file 2: Figure S1). Yet, this was significantly reduced in the HF + CON + PRO and not measurable in the HF + DEP + PRO group (Additional file 3: Figure S2), suggesting that the microbiome plays a role in IL-1 inflammatory response.

Tumor necrosis factor-alpha (TNF-α) is an important mediator of inflammatory response with involvement in inflammatory bowel disease and CVD [26, 56, 57]. In this study, DEP exposure resulted in an increase in TNF-α in HF diet mice only, showing that dietary stress on the microbiome exacerbates the DEP exposure–response. Moreover, we found that probiotic treatment reduced TNF-α in HF + DEP + PRO group compared to HF + DEP, confirming that alterations in the gut microbiome contribute to increased circulating TNF-α. The abundance of Proteobacteria followed the same trend as TNF-α in the current study. This agrees with a previous study that reported rats on a HF diet had increased serum TNF-α, serum LPS, and enriched abundance of Proteobacteria in the colon [58]. Proteobacteria are gram-negative bacteria and a possible source of circulating LPS resulting in LPS stimulation of TNF-α production by activated immune cells or other TNF-α expression cell types [59]. Furthermore, IL-1, TNF-α, and LPS are known regulators of G-CSF, which is a pro-inflammatory cytokine involved in CVD[60]. We observed that G-CSF is significantly elevated in our LF + DEP group, yet IL-1α and TNF-α are decreased and unchanged, respectively. However, LPS is also elevated in this group; therefore, it is plausible that in the LF + DEP animals, LPS is promoting upregulation of G-CSF.

IL-15 is a pleiotropic cytokine that has plays a vital role in immune regulation and stimulates natural killer (NK) cells. In contrast, G-CSF is known to inhibit natural killer (NK) cells. Interestingly, in our study, we found an increase in G-CSF and IL-15 in the LF + DEP group. NK cells are known to become activated during LPS stimulus. Therefore, we suspect that in LF + DEP animals, LPS is stimulating IL-15 and immune cell activated NK cells, and G-CSF is responding to the increase in NK cell activation. IL-3, also referred to as multipotential-CSF, is a T-helper-activated cytokine important in defense against pathogens and has been shown to work in conjunction with G-CSF to mediate the recruitment of peripheral blood progenitor cells from the bone marrow to circulation [61]. In the current study, IL-3 was exposure-dependent, although it was decreased in LF + DEP and increased in HF + DEP animals. This could be due to a compensatory response, as G-CSF is significantly elevated in LF + DEP. Notably, we observed a significant decrease in IL-3 with probiotic treatment. We speculate that the anti-inflammatory effects of *Bifidobacterium* and/or *Lactococcus* are responsible for the overall reduction of IL-3 in these groups [37, 40, 62].

LIF is an IL-6 pleiotropic cytokine that is mediated through specific cell-surface receptors and has an important role in immune response [63]. Due to its inhibitory function, a reduction in circulating LIF is associated with immune cell differentiation, suggesting HF diet and DEP exposure in our study resulted in LIF-mediated immune cell response. Although further investigation is needed, macrophage stimulation in the vasculature could account for decreased circulating LIF. Probiotic treatment did not alter circulating LIF in HF + DEP animals.

When comparing the microbiome of the HF diet animals, we observed that *Lactococcus* is expanded with probiotic treatment. *Lactococcus lactis* produces anti-inflammatory compounds and has been shown to stimulate the immune response in the gut and reduce overall inflammation associated with consumption of a HF diet [62, 64]. While the anti-inflammatory effects of *Lactococcus* are likely multifaceted, there is a direct correlation with HF diet, probiotic treatment, and the reduction in IL-13, MIP-2, and RANTES. IL-13 is an immunoregulatory cytokine associated with chronic inflammation and allergic response by IgE activation. It has been studied in obesity, metabolic syndrome, and colitis, and its activity in the lung and air pollution has been established [65, 66]. In our study, we see an elevated concentration of IL-13 in the plasma of HF diet mice, which was attenuated through probiotic treatment. MIP-2 is a major pro-inflammatory chemokine responsible for protection against pathogens and tissue damage by leukocyte recruitment [67]. RANTES, also known as CCL5, is a pleiotropic chemokine that signals immune cell infiltration to sites of inflammation or damage and is implicated in intestinal barrier damage [68]. Both MIP-2 and RANTES have been associated with obesity [69–71]. In our study, we saw a significant increase in MIP-2 with HF diet and a significant decrease in MIP-2 and RANTES with probiotic treatment. While further investigation of RANTES and MIP-2 signaling and adiposity in these animals is merited, it is plausible that microbial profiles and probiotics play a role in signaling.

Considering the systemic inflammatory response observed in the HF diet and DEP exposed mice, we decided to explore early markers of CVD within the heart. Leukocyte transmigration between endothelial cells is an important feature of inflammatory events, both systemically and in the heart. ICAM-1 is a cell surface receptor on endothelial cells that mediates T cell recruitment and adhesion of leukocytes during cardiovascular stress and has been indicated in endotoxemic cardiac dysfunction by cytokine activation [72, 73]. In our study, we observed a significant increase in sICAM-1 in the HF + DEP group, compared to HF + CON, which also displayed increased circulating TNF-α. While more investigation is warranted, early involvement of TNF-α in the circulation may be associated with the observed increase of sICAM. In agreement with this premise, we observed a decrease in sICAM-1, associated with decreased TNF-α, in the HF + DEP + PRO group. We also measured PECAM-1, an adhesion protein expressed in endothelial cells that mediates leukocyte migration between endothelial cells and, like sICAM-1, is indicated in endotoxemia [74, 75]. A reduction of PECAM-1 in probiotic-treated mice coincides with an observed reduction in circulating LPS in these animals. Soluble P-selectin (sP-selectin) is released from platelets and is a potential marker of platelet activation indicated in cardiovascular events [76]. We observed an increase of sP-selectin in HF + DEP hearts compared to LF + DEP. Interestingly, we found a significant increase in the concentration of sP-selectin in the hearts from the HF + CON + PRO group. While data is lacking related to sP-selectin and gut microbiome or probiotics, one study found a reduction of P-selectin in the colon following *Lactobacillus reuteri* administration in rats [77]. We recognize that measuring soluble P-selectin versus P-selectin may be a limitation to this data, as we used whole heart homogenate tissue instead of measuring in plasma as a marker for circulating platelet activation.

PAI-1 is an important serine protease inhibitor that plays a vital role in acute and chronic fibrinolysis in cardiovascular and pulmonary diseases, as well as obesity and metabolic disorders [78]. PAI-1inhibitors have been shown to effectively reduce HF diet-induced obesity, suggesting a link between HF diet and PAI-1 signaling [79]. A recent study found that G-CSF downregulated intracellular PAI-1 in hematopoietic stem cells by a complex signaling pathway involving transforming growth factor (TGF)-β [80]. DEP exposure resulted in a decrease in PAI-1 with LF diet only, where we also observed a significant increase in G-CSF between these groups. We speculate that inflammatory cytokine involvement in this study results in the downregulation of PAI-1 with exposure, while HF diet maintains an increased PAI-1.

Thrombomodulin is an endothelial thrombin receptor that acts as an anticoagulant by inhibiting the thrombin coagulation and downregulating fibrinolysis and inflammation [81]. The role of inhaled PM on blood coagulation has been established [82]. Our current study showed a significant decrease in thrombomodulin with DEP exposure, regardless of diet, which is correlated with increased inflammation and procoagulant state. With our probiotic treatment groups, we found no significant decrease with DEP exposure; however, there was a significantly decreased baseline concentration of thrombomodulin within the heart of probiotic groups. Interestingly, a study using the same Ecologic Barrier Probiotic formulation found that thrombomodulin was significantly decreased in obese postmenopausal women in a 12-week clinical, which is in agreement with the observed reduction of thrombomodulin within our probiotic treated mice [83].

Collectively, our study results show significant alterations in the gut microbial profiles and diversity, as well as systemic inflammatory and cardiovascular responses following a 30-day DEP exposure study. However, these alterations may occur differently at distinct time points due to the dynamic nature of the gut microbiome and its continual responsiveness to environmental changes and homeostatic responses with the host. For this reason, further investigation of DEP exposure ± diet permutations at both acute and chronic exposure time points is warranted. We chose OA exposure to DEP instead of whole-body inhalation to eliminate ingestion exposure via oral cavity, fur grooming, or food. This exposure approach allowed for specifically investigating the effects of inhaled PM from the lungs; however, it is also a noted limitation of the study in-so-much-that OA exposure is not consistent with nasal inhalation more relevant to human exposure scenarios. Moreover, we cannot accurately assess the amount of PM material that was introduced to the GI tract via mucociliary clearance following the OA exposure. Further studies are necessary to determine the degree to which the direct effects of cleared PM versus indirect contributions of translocated PM or systemic inflammatory signaling molecules are contributing to the observed effects on the gut and microbiome profiles.

One goal of the current study was to determine if probiotic-treatment could prevent the DEP exposure ± HF diet mediated alterations in microbiota profiles and subsequent detrimental systemic and cardiac outcomes. Our results revealed that the microbiome plays a vital role in mediating these systemic inflammatory responses following a 30-day exposure/diet protocol. Previous ambient PM and vehicle exhaust exposure studies have described resulting alterations in the gut microbiome and systemic inflammation [9, 16]; however, the effects of traffic-generated PM, such as DEP, on these outcomes is not as well characterized. The dose of DEP used in the current study is higher than that typically experienced in an ambient environmental scenario, representing approximately 10 μg/mouse/day, which, when adjusted in mice, is estimated to be nearly 40-fold higher than alveolar deposition resulting from a 24 h inhalation exposure of 100 μg/m^3^ in humans [84]. However, we chose this dose based on previously characterized effects in the lung and cardiovascular system [84]. We also recognize that the DEP utilized for this study is not necessarily representative of all diesel engine-generated PM. Nevertheless, this study provides preliminary information on the outcomes of subacute DEP exposure and diet-mediated alterations on gut microbial profiles and associated systemic inflammatory signaling pathways.

## Conclusion

Our findings demonstrate that inhaled DEP exposure alters the gut microbial profiles and diversity, increases systemic inflammatory response, and is implicated in the etiology of the early stages of CVD. The apparent reduction in *Bifidobacterium* and expansion of *Akkermansia* and *Escherichia* in the gut with HF diet and DEP exposure is of particular importance in further understanding of how air pollution influences gastrointestinal disease, as well as its implications in contributing to inflammation and CVD (Fig. 16). The use of probiotics in this study proved to be pivotal in understanding the influences that the microbiome has on systemic and CVD effects following DEP exposure ± HF diet. Thus, our current findings serve as a foundation for future studies to investigate the mechanisms involved in gut-heart axis signaling and provides insight into the potential therapeutic use of probiotics for microbiome manipulation and prevention or treatment of diseases associated with systemic inflammation, including CVD.

## Methods

### Animal care and diet

Four-to-six-week-old C57Bl/6 wild-type male mice (Taconic, C57BL/NTac) were fed ad libitum either a low-fat diet (LF) consisting of 10% fat and 17% sucrose (Research Diets #D124505H, *n* = *24*) or a HF diet consisting of 45% fat and 17% sucrose (HF, Research Diets #D12451, *n* = *48*) during the entire course of the study. Mice were co-housed 4 per standard shoebox cage with a barrier filter lid in a humidity and temperature-controlled environment on a 12-h light and 12-h dark cycle. Animals were acclimated for 1 week, then placed on their respective controlled low-fat or high-fat diet and/or probiotic treatment 3 days prior to first exposure. Diet and probiotics were maintained throughout the course of the study. All animal procedures were reviewed and approved by the University of North Texas IACUC and follow the *Guide for the Care and Use of Laboratory Animals* (NIH Publication No. 85-23, rev. 1996). The authors note that the animals in this exposure and probiotic treatment study are the same animals from Daniel et al. [46].

### Exposures

Mice were randomly assigned to be exposed via oropharyngeal aspiration (OA) to 35 μg diesel exhaust particles (DEP) purchased from National Institute of Standards and Technology (NIST, Standard reference material #2975) suspended in 35 μl 0.9% sterile saline (DEP, *n* = *36*), or sterile saline only (CON, *n* = *24*) twice a week for 4 weeks. The NIST DEP #2975 particle size was previously analyzed by Miller at el., which reported a mean DEP particle size of 257 ± 46 nm [84], and a mean diameter (volume distribution) of 31 ± 0.6 μm, as reported in the certificate analysis by NIST. DEP mixtures were suspended in saline and then sonicated for 5 min immediately prior to the start of dosing for each exposure day. Furthermore, the DEP suspension was vortexed for 1 min before each animal exposure to ensure homogeneity was maintained throughout the exposure period. All chemical constituents for the chosen DEP material is characterizied and ceritified by NIST. We previously reported the measured the levels of LPS within the DEP, which was determined to be negligible [46]. For OA, mice were anesthetized with 2% Isoflurane (Butler Schein Animal Health), then suspended on a surgery board (rodent intubation stand; Biolite RIS 100), at approximately 60° angle. The tongue was gently pulled to the side using forceps, suspended DEP or saline was administered into the distal portion of the oropharyngeal cavity while the nose was gently closed until the suspension was aspirated. Following aspiration, the mouse was removed from the surgery board, placed on a warming pad, and monitored until fully recovered from anesthesia. The rationale for choosing OA was two-fold: (1) OA exposure route simulates the route particulate matter would take from the oropharynx region into the lungs, including contact with the mucociliary escalator, which is likely an important source for ingested PM after inhalation exposure and effects on the gut and/or resulting microbial profiles, and (2) compared to whole chamber inhalation models, OA exposure limits the amount of particulate ingested during grooming and/or eating.

### Probiotic treatment

A subset of mice on the HF diet were dosed with 0.3 g/day of probiotic (~ 7.5 × 10^8^ CFU/day) Ecologic® Barrier 849 (Winclove Probiotics) in drinking water (PRO, *n* = *12*) throughout the exposure study. Probiotic formulation is a proprietary probiotic blend consisting of nine bacterial strains: *Bifidobacterium bifidum* W23, *B. lactis* W51, *B. lactis* W52, *Lactobacillus acidophilus* W37, *L. brevis* W63, *L. casei* W56, *L. salvarius* W24, *Lactococci lactis* W19, and *Lc. lacis* W58 (‘‘Ecologic® Barrier”, Winclove Probiotics B.V., Amsterdam, The Netherlands) in a carrier matrix of maize starch, maltodextrin, and minerals. Probiotics were provided in low-drip water bottles and measured daily to determine the average consumption of probiotics per cage. Probiotics were changed daily between 4 and 6 pm in cleaned bottles. Table 5 shows the exposure, diet, and probiotic treatment groups names used throughout the manuscript.

**Table 5:** Diet, exposure, and probiotic treatment groups

	Diet	Exposure	Probiotic treatment	Group
Male C57Bl/6 4–6 weeks old	Low fat	Saline	No	LF + CON (n = 12)
Male C57Bl/6 4–6 weeks old	Low fat	DEP	No	LF + DEP (n = 12)
Male C57Bl/6 4–6 weeks old	High fat	Saline	No	HF + CON (n = 12)
Male C57Bl/6 4–6 weeks old	High fat	DEP	No	HF + DEP (n = 12)
Male C57Bl/6 4–6 weeks old	High fat	Saline	Yes	HF + CON + PRO (n = 12)
Male C57Bl/6 4–6 weeks old	High fat	DEP	Yes	HF + DEP + PRO (n = 12)
Total mice				72

DEP, diesel exhaust particles; CON, saline controls, HF, high-fat diet; LF, low-fat diet; PRO, probiotic treatments

### Tissue collection

Mice were anesthetized with Euthasol and euthanized by exsanguination via cardiac puncture within 24 h of the final DEP exposure. Blood was collected in heparinized tubes and centrifuged at 4 °C at 15,000 g for 10 min. Supernatant plasma was collected and stored at − 80 °C. The whole heart was removed, snap-frozen in liquid nitrogen, and stored at − 80 °C for further analysis. The stomach, small intestine, and cecum were immediately excised and weighed. Small intestinal luminal contents were flushed with sterile PBS, collected, and snap-frozen in liquid nitrogen then stored at − 80 °C. The small intestine was dissected into three portions: duodenum, jejunum, and ileum, which were snap-frozen then stored in − 80 °C for later analysis. Plasma samples were treated HemogloBind (Biotech Support Group LLC, East Brunswick, NJ #HO145), which is a bead-based product that removes hemoglobin without interacting with proteins or biomarkers. HemogloBind was added to plasma samples (1:1 ratio), vortexed for 30 s, inverted for 10 min, and centrifuged for 2 min at 9000 RPMs. The supernatant was removed using wide-bored pipette tips and stored at − 80 °C for downstream analysis.

### DNA extraction

Bacterial DNA isolation was performed on combined small intestine tissue and luminal contents using ZR Fecal DNA miniprep (Zymo Research, Irvine, CA) following the manufacturers protocol. Two-centimeter portions of the duodenum, jejunum, and ileum were excised, combined with luminal content pellet (following centrifugation at 15,000 g for 15 min at 4 °C), then homogenized using bead beater. DNA quantification and quality checks were performed using Shimadzu BioSpec-nano (Shimadzu Corporation, Japan).

### Illumina MiSeq

Following DNA extraction, qPCR, and quality checks, samples were submitted to Salient Genomics (Krum, TX) for 16S rRNA gene V4 region sequencing using Illumina MiSeq 2 platform. Samples were prepared and amplified using the Illumina 16S rRNA gene metagenomic library prep guide (Illumina, #15044223 Rev. B). Samples were amplified by PCR and PCR product was cleaned using AmPure XP paramagnetic beads (Beckman Coulter, Indianapolis, IN). A second round of PCR to label sample DNA fragments with indices was done using TruSeq UDI kit (Illumina, San Diego, CA) with 10 base pairs each. Second PCR product was cleaned using AmPure XP paramagnetic beads (Beckman Coulter, Indianapolis, IN). Concentrations were determined for each sample using Qubit HS DNA assay (cat.# Q32851, Invitrogen, Waltham, MA) on Qubit Fluorometer (ThermoFisher, Waltham, MA) and the length of samples was verified using the Agilent Bioanalyzer High Sensitivity DNA assay (Agilent, Santa Clara, CA). Samples were then diluted and run on Illumina MiSeq 2 benchtop platform. Secondary analysis was performed using MiSeq Reporter software and fastq files were generated for downstream analysis.

### Bioinformatics

16S rRNA gene sequencing reads (in fastq format) were analyzed using methods previously established by our lab [85]. Downstream statistical analysis of the data was performed to determine alpha diversity analysis (diversity within the sample) by generating the plots for Chao 1 and ACE index (for species richness), Shannon index (for species diversity), and evenness index (for species evenness). Beta diversity (diversity between the samples) analysis was performed by generating Unifrac weighted and unweighted principal coordinate analysis (PCoA) plots (Lozupone and Knight 2005). Note that the samples that could skew the alpha–beta diversity analysis because of their sequence counts were removed from the downstream analysis. The diversity analysis was performed using phyloseq package in the R environment [86]. The statistical significance of these outcomes, including the diversity estimation, was inferred using AMOVA and ANOVA in Mothur [87].

### Taxonomic classification and relative abundance

Amplicon sequence data files were fastq formatted and the taxonomic classification of sequences was performed using the Illumina-curated version of Greengenes database and the 16S rRNA gene Metagenomics application within Illumina BaseSpace (version 1.0.1). Default parameters were used. To determine relative abundance, the upper 8 most abundant phyla and upper 20 most abundant genera were used when comparing LF + CON:LF + DEP:HF + CON:HF + DEP and HF + CON:HF + DEP:HF + CON + PRO:HF + DEP + PRO. Each sample’s OTU counts were normalized by total OTU counts for each phylum and genus. Outliers were removed using ROUT (Q = 1%). Relative abundance was determined by fraction of total for the eight most abundant phyla and twenty most abundant genera in GraphPad Prism 9. To represent changes with DEP exposure, we determined the percent change between DEP exposed and their respective controls (Figs. 1, 8). Relative abundance and percent change are determined using two different analyses, where LF + CON, LF + DEP, HF + CON, and HF + DEP uses the most abundant phyla and genera within the four groups and where HF + CON, HF + DEP, HF + CON + PRO, and HF + DEP + PRO use the most abundant phyla and genera within these four groups.

### Probiotic quantification

Paired-end reads were merged using PEAR (https://doi.org/10.1093/bioinformatics/btt593). Sequencing analysis was conducted using the VSEARCH open-source pipeline (https://doi.org/10.7717/peerj.2584). Raw sequence data was filtered, dereplicated and screened at default parameters. The sequences were then clustered into operational taxonomic units (OTUs) based on 99% similarity identity. To determine the presence of probiotics in the sample, the V4 regions 16S rRNA gene bacterial samples were aligned as queries via global alignment in VSEARCH against a database constructed of the 16S rRNA gene sequences for the probiotics used in the study. The relative abundance of each probiotic was determined by dividing the total count for each given probiotic by the total number of screened reads for each sample. The probiotic strains *Bifidobacteria lactis* W52 and W51 have a 99.8% similarity within the V4 region and *Lactococcus lactis* W19 and W58 have 100% similarity within the V4 region, therefore we were unable to differentiate these individual strains in the samples. These strains were grouped for analysis purposes to give 7 bacterial strains for probiotics quantification.

### Circulating LPS

Circulating LPS levels were measured in plasma using Pierce Chromogenic endotoxin Quant Kit (ThermoFisher, Waltham, MA) with a 1:100 dilution in endotoxin-free water following the manufacturer's protocol. Samples were run in duplicate and values were determined by known standard curve. Results are expressed as EU/mL. *n* = 5–6 samples per group for analysis. Samples measured out of standard curve were removed from the analysis.

### Cytokine/chemokine biomarkers

To determine systemic inflammation, plasma concentrations of G-CSF, IFNγ, IL-1α, IL-1β, IL-2, IL-3, IL-9, IL-12(p20) IL-13, IL-15, LIF, LIX, MCP-1, M-CSF, MIG, MIP-2, RANTES, and TNF-α were measured using Mouse Cytokine/Chemokine Magnetic Bead Panel (Milliplex, St. Louis, MO) following manufacturers protocol for plasma samples. Samples were not further diluted following pretreatment when HemogloBind (dilution factor following treatment of 1.75). Samples were run on Luminex FLEXMAP 3D® instrument system with xPONENT software (EMD Millipore; St. Louis, MO) following the manufacturer's recommendation. Median fluorescent intensity data was used to determine unknown concentrations in Milliplex Analyst Software (EMD Millipore, St. Louis, MO). Samples measuring outside of the standard curve were removed from the analysis (*n* = 3–6). For supplementary data *n* = 2–8 and groups with all measurements below the standard curve are represented as no data (ND).

### Cardiovascular protein extraction

For protein extraction of cardiac tissue, 20–40 mg of heart (atria and ventricles) tissue was placed in lysis buffer (0.01 g bovine serum albumin, 5 μl of Triton-X 100, 5 μl protease inhibitor cocktail (Calbiochem, MilliporeSigma, St. Louis, MO, cat. #535140), and 990 μl PBS) and homogenized in bead beater for 15 min. Next, samples were incubated at 4 °C on a rocker for 15 min, then centrifuged at 15,000 RPM for 5 min at 4 °C. The supernatant was removed and frozen at − 20 °C. Samples were then filtered in 0.22 μm syringe-driven filter unit (Millipore, St. Louis, MO, cat. #SLGP033RS). Samples were checked for purity and protein quantification using Take3™ Micro-Volume Plate (BioTek, Winooski, VT) read on Epoch™ Microplate Spectrophotometer with Gen5 Software (BioTek, Winooski, VT).

### Cardiovascular disease biomarkers

To determine the presence of cardiovascular disease biomarkers in the heart, cardiac tissue protein concentrations of sICAM-1, PECAM-1, sP-selectin, PAI-1, and thrombomodulin were determined using Mouse CVD Magnetic Bead Panel 1 (Milliplex, St. Louis, MO) following manufacturers protocol for tissue culture supernatant. Tissue lysis buffer was used used for matrix solution for samples. Samples were run on Luminex FLEXMAP 3D® instrument system with xPONENT software (EMD Millipore; St. Louis, MO) following the manufacturer's recommendation. Median fluorescent intensity data was used to determine unknown concentrations in Milliplex Analyst Software (EMD Millipore, St. Louis, MO). Sample concentrations were normalized to total protein concentration prior to statistical analysis. *n* = 4–6. Samples were removed from analysis because of (1) red coloration of sample due to red blood cell lysis, or (2) sample biomarker concentrations were not within standard curve range or below the measurable threshold.

### Statistical analysis

Where not otherwise indicated, data were analyzed by two-way ANOVA with Sidak-Holm multiple comparison all-pairwise test using GraphPad Prism 9. Two-way ANOVA was used to determine the relationship between two independent factors—diet and exposure to inhaled DEP and the interaction between diet and exposure. For the probiotic studies, since all animals were on a high fat diet, a two-way ANOVA was used to determine the statistical relationship between the two factors of exposure and probiotic treatment, as well as the interaction between exposure and probiotic treatment. The Results section shows the corresponding *p* values and F values for each pairwise interaction. Outliers were determined and removed using ROUT method with a Q value of 1% in GraphPad Prism 9. Data are expressed as mean ± SEM and a *p* < 0.05 was considered statistically significant. Special notations have been made for data with *p* < 0.06 and *p* < 0.07 within the Results sections and figures.

## Supplementary Information

**Additional file 1**: **Table S1**. Mean, standard deviation, and standard error of the mean for the most abundant phyla across exposure, diet, and probiotic treatment groups.

**Additional file 2**: **Figure S1**: Mean circulating inflammatory cytokines: (A) IFN-γ, (B) IL-1β, (C) IL- 2, (D) IL-9, (E) IL-12(p40), (F) LIX, (G) MCP-1, (H) M-CSF, and (I) MIG measured in plasma in C57Bl/6 male mice exposed to inhaled diesel exhaust particles (DEP- 35 μg PM) or saline control (CON) twice a week for four weeks on either low-fat (LF) or high-fat (HF) diet. Data were analyzed by two-way ANOVA. n=2-5.

**Additional file 3**: **Figure S2**: Mean circulating inflammatory cytokines: (A) IFN-γ, (B) IL-1β, (C) IL- 2, (D) IL-9, (E) IL-12(p40), (F) LIX, (G) MCP-1, (H) M-CSF, and (I) MIG measured in plasma in C57Bl/6 male mice on high-fat (HF) diet exposed to diesel exhaust particles (DEP- 35 μg PM) or saline control (CON) twice a week for four weeks with a subset of mice given 0.3 g/day (~ 7.5 × 107 cfu/day) of Ecologic® Barrier probiotics (PRO) in the drinking water throughout the exposures. (B) IL-1β data was measured by t-test due to no measurable data for HF+DEP+PRO group *p<0.05 compared to HF+CON, †p<0.05 compared to HF+DEP by t-test. Data is depicted ± SEM and was analyzed by two-way ANOVA. ND indicates samples were below measurable threshold or below the standard curve. n=2-5.

## Data Availability

All datasets used and/or analyzed during the current study are available from the corresponding author on reasonable request.

## Abbreviations

CON: Control
CVD: Cardiovascular disease
DEP: Diesel exhaust particulate matter
HF: High-fat diet
IFN-γ: Interferon-gamma
IL: Interleukin
G-CSF: Granulocyte colony-stimulating factor
LIF: Leukocyte inhibitory factor
LIX: LPS induced CXC
LF: Low-fat diet
LPS: Lipopolysaccharide
M-CSF: Macrophage colony-stimulating factor
MCP: Monocyte chemoattractant protein
MIG: Monokine induced by gamma interferon
MIP: Macrophage inflammatory protein
PAI: Plasminogen activator inhibitor
PECAM: Platelet endothelial cell adhesion molecule
PM: Particulate matter
RANTES: Regulated upon activation, normal T cell expressed and presumably secreted
sICAM: Soluble intracellular adhesion molecule
sP-selectin: Soluble platelet-selectin
TGF-β: Transforming growth factor beta
TNF-α: Tumor necrosis factor-alpha
