# Supplementary material for: Inhaled diesel exhaust particles result in microbiome-related systemic inflammation and altered cardiovascular disease biomarkers in C57Bl/6 male mice
Source: Part Fibre Toxicol. 2022 Feb 9;19:10. doi: 10.1186/s12989-022-00452-3 (PMC8827295; doi:10.1186/s12989-022-00452-3)
Supplement: Supplementary file 1 — Additional file 1: Table S1. Mean, standard deviation, and standard error of the mean for the most abundant phyla across exposure, diet, and probiotic treatment groups. [file 12989_2022_452_MOESM1_ESM.pdf]

Supplementary Table 1: Mean, standard deviation, and standard error of mean for most abundant phyla across exposure, diet, and probiotic treatment groups.

		LF+CON	LF+DEP	HF+CON	HF+DEP	HF+CON+PRO	HF+DEP+PRO
**Firmicutes**	Mean	50.980	60.380	68.660	62.040	62.600	62.060
	SD	20.230	14.700	15.440	27.570	16.380	10.270
	SEM	7.151	5.196	5.459	10.420	6.192	3.881
**Proteobacteria**	Mean	28.030	21.890	19.930	34.710	23.990	24.350
	SD	27.060	22.130	14.490	27.280	21.510	15.620
	SEM	9.567	7.824	5.124	10.310	8.130	5.902
**Actinobacteria**	Mean	18.060	6.548	9.380	1.347	11.480	10.530
	SD	12.040	8.200	9.033	0.939	5.481	5.413
	SEM	4.259	2.899	3.194	0.355	2.072	2.046
**Verrucomicrobia**	Mean	2.469	5.355	1.101	0.275	1.333	1.138
	SD	3.175	4.247	1.749	0.314	2.352	1.499
	SEM	1.122	1.501	0.618	0.119	0.889	0.567
**Bacteroidetes**	Mean	0.131	5.231	0.566	0.990	0.350	1.095
	SD	0.071	13.420	0.821	1.809	0.316	1.196
	SEM	0.025	4.743	0.290	0.684	0.120	0.452
**Deferribacteres**	Mean	0.113	0.375	0.066	0.309	0.018	0.136
	SD	0.277	0.967	0.154	0.719	0.029	0.231
	SEM	0.098	0.342	0.054	0.272	0.011	0.087
**Thermi**	Mean	0.045	0.056	0.202	0.176	0.119	0.489
	SD	0.043	0.047	0.331	0.185	0.105	1.033
	SEM	0.015	0.017	0.117	0.070	0.040	0.390
**Tenericutes**	Mean	0.050	0.076	0.037	0.017	0.023	0.130
	SD	0.042	0.057	0.048	0.018	0.032	0.275
	SEM	0.015	0.020	0.017	0.007	0.012	0.104

DEP, diesel exhaust particles; CON, saline controls; LF, low fat diet; HF, high fat diet; PRO, probiotic treatment; SD, standard deviation; SEM, standard error of mean.
