# Supplementary figures and images for: Inhaled diesel exhaust particles result in microbiome-related systemic inflammation and altered cardiovascular disease biomarkers in C57Bl/6 male mice
Source: Part Fibre Toxicol. 2022 Feb 9;19:10. doi: 10.1186/s12989-022-00452-3 (PMC8827295; doi:10.1186/s12989-022-00452-3)

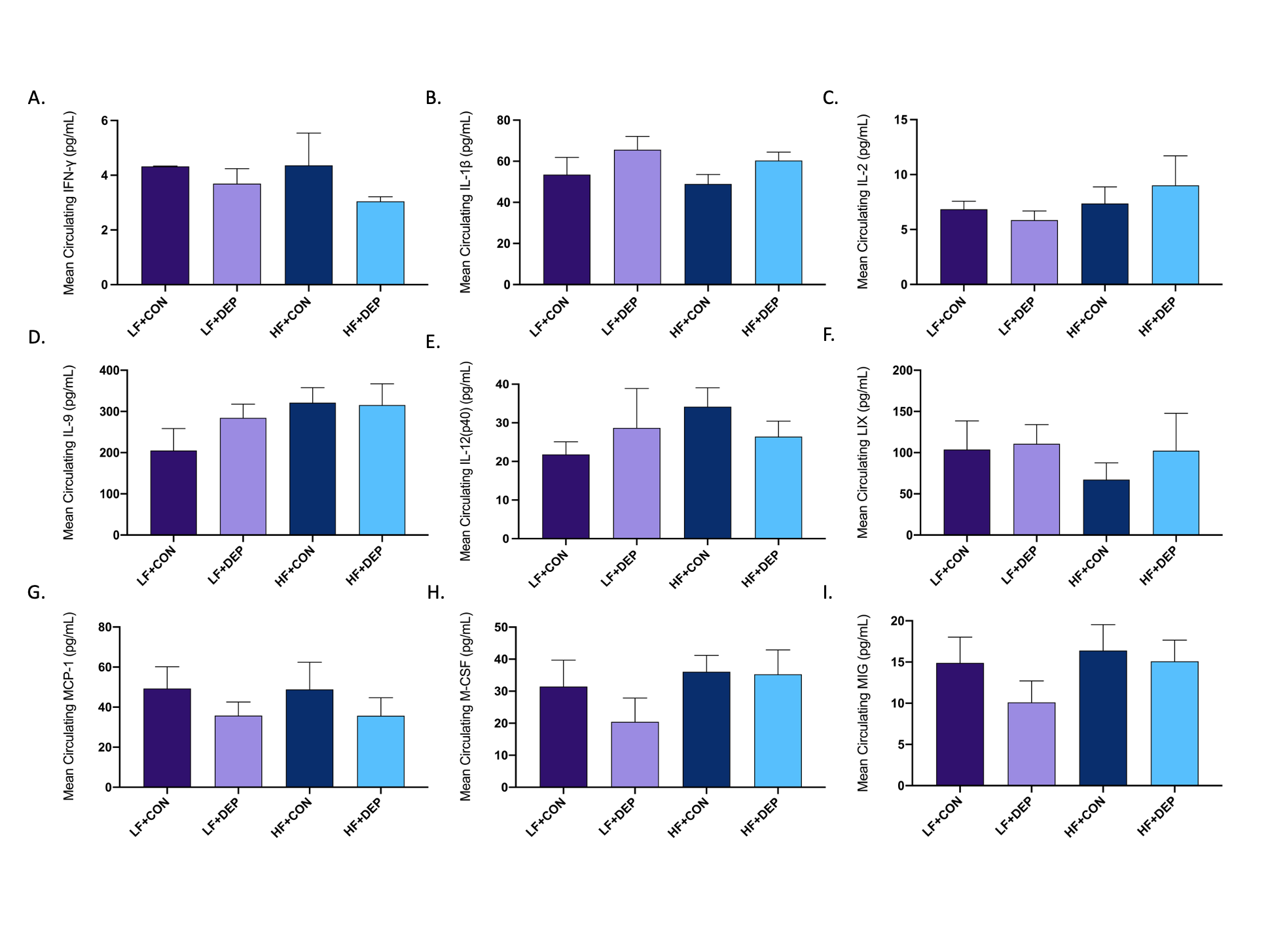

Supplement: Supplementary file 2 — Additional file 2: Figure S1: Mean circulating inflammatory cytokines: (A) IFN-γ, (B) IL-1β, (C) IL- 2, (D) IL-9, (E) IL-12(p40), (F) LIX, (G) MCP-1, (H) M-CSF, and (I) MIG measured in plasma in C57Bl/6 male mice exposed to inhaled diesel exhaust particles (DEP- 35 μg PM) or saline control (CON) twice a week for four weeks on either low-fat (LF) or high-fat (HF) diet. Data were analyzed by two-way ANOVA. n=2-5. [file 12989_2022_452_MOESM2_ESM.tif]

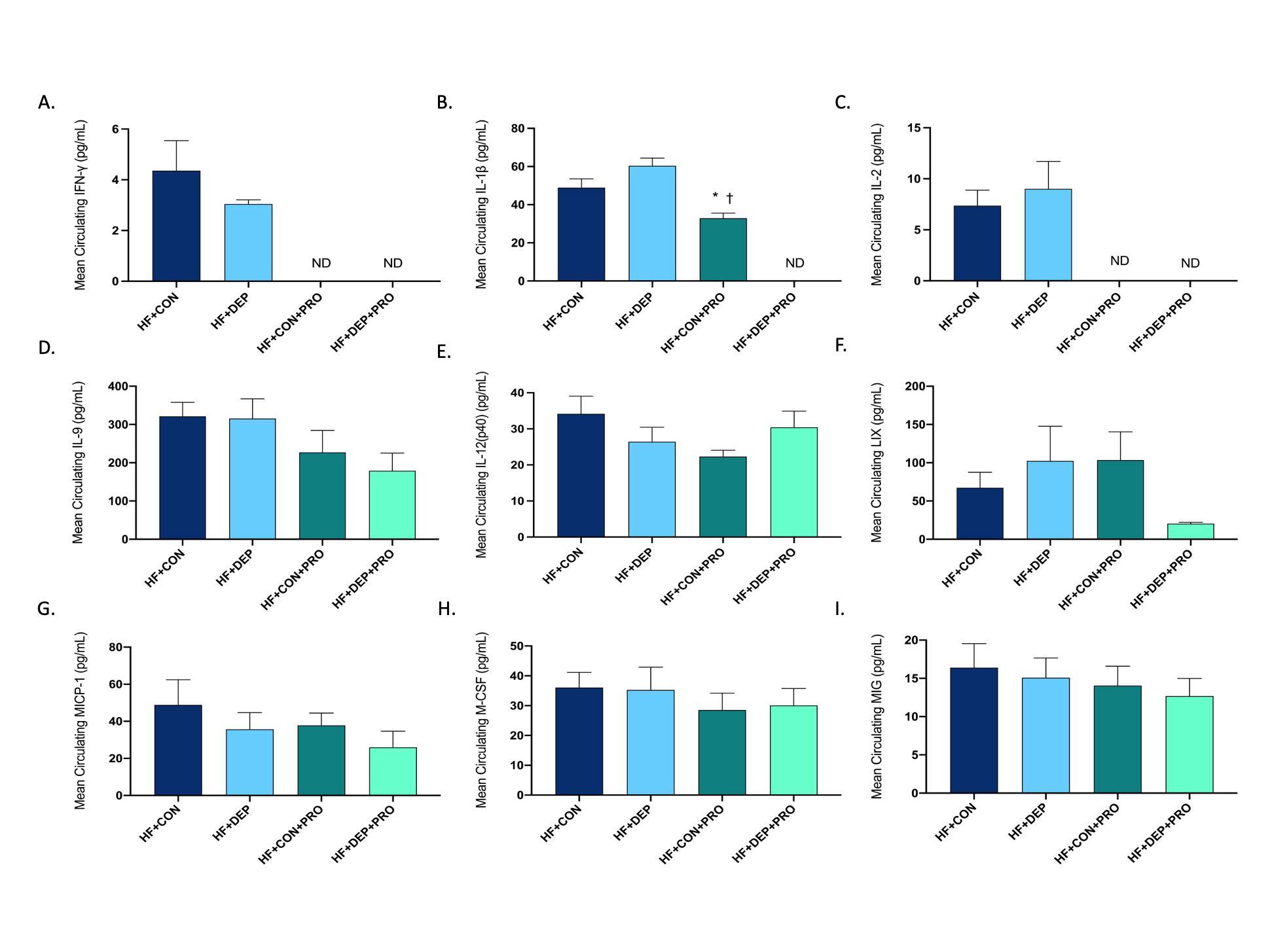

Supplement: Supplementary file 3 — Additional file 3: Figure S2: Mean circulating inflammatory cytokines: (A) IFN-γ, (B) IL-1β, (C) IL- 2, (D) IL-9, (E) IL-12(p40), (F) LIX, (G) MCP-1, (H) M-CSF, and (I) MIG measured in plasma in C57Bl/6 male mice on high-fat (HF) diet exposed to diesel exhaust particles (DEP- 35 μg PM) or saline control (CON) twice a week for four weeks with a subset of mice given 0.3 g/day (~ 7.5 × 10^7^ cfu/day) of Ecologic® Barrier probiotics (PRO) in the drinking water throughout the exposures. (B) IL-1β data was measured by t-test due to no measurable data for HF+DEP+PRO group *p<0.05 compared to HF+CON, †p<0.05 compared to HF+DEP by t-test. Data is depicted ± SEM and was analyzed by two-way ANOVA. ND indicates samples were below measurable threshold or below the standard curve. n=2-5. [file 12989_2022_452_MOESM3_ESM.tif]
